# Meta-Analysis of Common and Differential Transcriptomic Responses to Biotic and Abiotic Stresses in *Arabidopsis thaliana*

**DOI:** 10.3390/plants11040502

**Published:** 2022-02-12

**Authors:** Yaser Biniaz, Aminallah Tahmasebi, Alireza Afsharifar, Ahmad Tahmasebi, Péter Poczai

**Affiliations:** 1 Plant Virology Research Center, Faculty of Agriculture, Shiraz University, Shiraz 7144113131, Iran; yser.biniaz@shirazu.ac.ir (Y.B.); afshari@shirazu.ac.ir (A.A.); 2 Department of Agriculture, Minab Higher Education Center, University of Hormozgan, Bandar Abbas 7916193145, Iran; a.tahmasbi@hormozgan.ac.ir; 3 Plant Protection Research Group, University of Hormozgan, Bandar Abbas 7916193145, Iran; 4 Institute of Biotechnology, Faculty of Agriculture, Shiraz University, Shiraz 7144113131, Iran; atahmasebi1@gmail.com; 5 Finnish Museum of Natural History, University of Helsinki, P.O. Box 7, FI-00014 Helsinki, Finland; 6 Faculty of Biological and Environmental Sciences, University of Helsinki, P.O. Box 65, FI-00065 Helsinki, Finland; 7 Institute of Advanced Studies Kőszeg (iASK), P.O. Box 4, H-9731 Kőszeg, Hungary

**Keywords:** *Arabidopsis thaliana*, biotic and abiotic stresses, transcriptome data, plant-stress interaction, plant response to multiple stresses

## Abstract

Environmental stresses adversely affect crop growth and yield, resulting in major losses to plants. These stresses occur simultaneously in nature, and we therefore conducted a meta-analysis in this study to identify differential and shared genes, pathways, and transcriptomic mechanisms involved in *Arabidopsis* response to biotic and abiotic stresses. The results showed a total of 436/21 significant up-/downregulated differentially expressed genes (DEGs) in response to biotic stresses, while 476 and 71 significant DEGs were respectively up- and downregulated in response to abiotic stresses in *Arabidopsis thaliana*. In addition, 21 DEGs (2.09%) were commonly regulated in response to biotic and abiotic stresses. Except for *WRKY45* and *ATXTH22*, which were respectively up-/down- and down-/upregulated in response to biotic and abiotic stresses, other common DEGs were upregulated in response to all biotic and abiotic treatments. Moreover, the transcription factors (TFs) bHLH, MYB, and WRKY were the common TFs in response to biotic and abiotic stresses. In addition, ath-miR414 and ath-miR5658 were identified to be commonly expressed in response to both biotic and abiotic stresses. The identified common genes and pathways during biotic and abiotic stresses may provide potential candidate targets for the development of stress resistance breeding programs and for the genetic manipulation of crop plants.

## 1. Introduction

Plants are continuously exposed to a wide range of adverse challenges under natural environmental conditions, which adversely affect plant growth, development, crop quality, and quantity. They are faced with various biotic stresses, including bacteria, fungi, viruses, herbivorous insects, and nematodes [1]. Moreover, abiotic stresses, such as salinity, drought, and extreme temperature, affect many physiological processes in plants [2]. The regulation of proper responses is crucial to reduce the damage caused by these stresses [3]. Various stresses initiate highly complex responses that lead to specific modifications at the transcriptome, cellular, and physiological levels. The plant stress response to adverse conditions includes complex interactions among their respective signaling pathways [4,5]. Environmental factors, such as drought, extreme temperature, and salinity, potentially alter the outcome of plant-pathogen interactions by modulating plant responses to the pathogen in question [6,7]. Abiotic stress may therefore lessen or elevate plant defense responses. On the contrary, certain pathogens may alter plant responses to abiotic stress factors. Evidence highlights the complex nature of interactions between biotic and abiotic stress responses [6,7,8]. The large amount of diverse omics datasets, such as genomic and transcriptomic data, of the model plant species *Arabidopsis thaliana* provides an opportunity to obtain information regarding the molecular mechanisms of stress responses [9,10]. In this context, systematic studies are pivotal to understanding the effects of conserved immunity responses to both biotic and abiotic stress conditions, which will provide guidance for improving crop resistance. Plant defense responses include cell wall reinforcement, reactive oxygen species generation, phytohormone production such as salicylic acid (SA), abscisic acid (ABA), jasmonic acid (JA), and ethylene (ET), and pathogenesis-related protein production, along with the development of a hypersensitive response [11]. To maintain the homeostasis required for a living organism to respond dynamically to stresses, a trade-off between plant growth and defense occurs in plants due to limited resources [6,12]. Functional molecular studies have investigated the interactions between biotic and abiotic stress signaling components by identifying a large number of genes regulated by various transcription factor (TF) types [13]. A better understanding of plant stress response mechanisms under various stresses can give us a better view for improving the worldwide food production [14]. Accordingly, to gain a broader perspective of the mechanisms that contribute to plant immunity, it is necessary to investigate genes at the pathway/genome levels rather than evaluating a single gene and learning more about the molecular mechanisms related to the cell wall, TFs, mitogen-activated protein kinases (MAPKs), reactive oxygen species (ROS) signaling pathways, hormone signaling, and to study genes encoding regulatory molecules, especially TFs [15,16]. Many efforts have been made to understand the molecular mechanisms of plant responses to stresses [17,18], whereas the effects of stress combinations remain poorly studied [6,19]. Studying the combination of various stresses is challenging. A vast and diverse set of genomic data has become publicly accessible with progress in biological high-throughput technology. Combining information from multiple existing studies facilitates obtaining a more accurate and comprehensive picture of plant defense responses and increasing reliable results [15,20,21,22]. In this study, we performed a large-scale meta-analysis based on publicly available gene expression data to explore the expression pattern of *Arabidopsis* pathways in response to biotic and abiotic stresses. However, relatively little information is available on the common signaling and transcriptomic responses to combined biotic/abiotic stresses. Therefore, we focused on how abiotic stresses affect plant molecular responses related to pathogens and how plants activate general stress responses to environmental stresses. A few other analysis methods, such as GO and Network analysis, were used to analyze the complexity of *Arabidopsis* response to biotic and abiotic stresses. The functions of the predicted stress-responsive TFs and microRNA (miRNA) are also discussed.

## 2. Results and Discussion

### 2.1. Identification of DEGs in Arabidopsis thaliana in Response to Biotic/Abiotic Stresses

**Figure 1:**
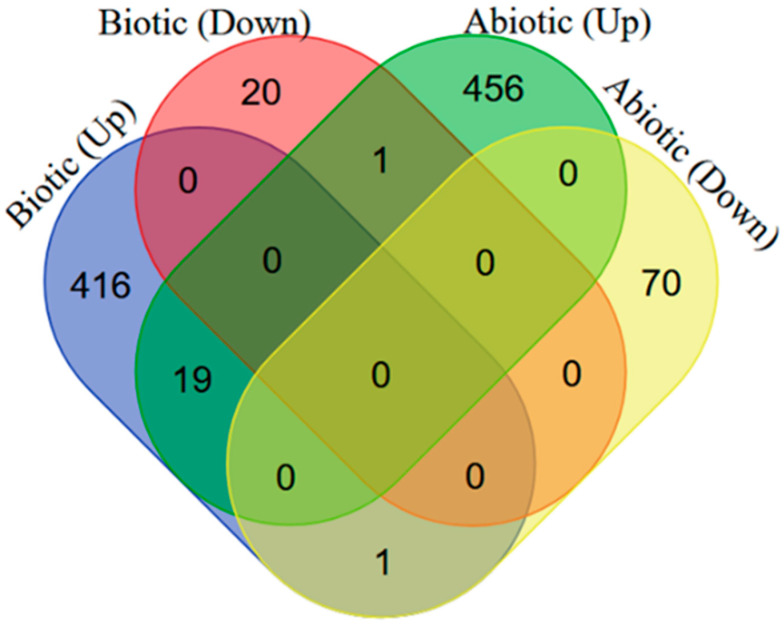
Identification of genes involved in biotic and abiotic stresses. Comparison of differentially expressed genes (DEGs) during abiotic and biotic stress responses. Four-way Venn diagrams showing co-occurrence and up-/downregulation of DEGs in response to various abiotic and biotic stresses.

Differential and common changes in gene expression of *A*. *thaliana* were investigated in response to biotic and abiotic stresses. We identified a total of 436/21 significant up-/downregulated DEGs in response to biotic stresses, while 476 and 71 significant differentially expressed genes (DEGs) were respectively up- and downregulated in response to abiotic stresses in *A. thaliana* (*p*-value < 0.01, |fold changes| >1). In addition, a set of 21 DEGs (2.09%) were commonly regulated between biotic and abiotic stresses. Except for two DEGs (AT3G01970; *WRKY45* and AT5G57560; *ATXTH22*) that were respectively up-/down- and down-/upregulated in response to biotic and abiotic stresses, other common DEGs (1.89%) were upregulated in response to all biotic and abiotic treatments (Figure 1).

Our results therefore highlighted that the most common DEGs in both biotic and abiotic stresses were upregulated. We conclude that a number of common pathways are induced by biotic and abiotic stresses. The common genes may play a role in the resistance against biotic and abiotic stresses and may be considered potential candidates in plant breeding programs. The DEGs including AT2G44890 (cytochrome P450) and AT1G09935 (phosphoglycerate mutase family protein) were the most upregulated ones in response to biotic stresses, while AT5G59720 (heat shock protein 18.2) and AT1G72660 (p-loop-containing nucleoside triphosphate hydrolases superfamily protein) were the highest upregulated DEGs in response to abiotic stresses. These genes showed the most upregulated expression in response to biotic and abiotic stresses. Previous studies have shown that cytochrome P450s play a key role in the biosynthesis and regulation of antioxidants, fatty acids, cell wall components, biopolymers, defense compounds, and phytohormones in plants [23,24]. These mediate plant defense responses and protect plants from biotic stresses including insects and diseases [25,26]. The expression of a phosphoglycerate mutase homologue in *Arabidopsis* is also reportedly regulated by hormones and induced by cyst and root-knot nematodes [27]. Heat shock protein 18.2 was highly expressed during abiotic stresses, which is in agreement with another study, and was found to be upregulated in response to heat, cold, and drought stresses [28,29]. Another highly expressed gene encoding a p-loop-containing nucleoside triphosphate hydrolase superfamily protein has been shown to play a role in salt tolerance in transgenic *A. thaliana* during abiotic stresses [30].

### 2.2. Gene Ontology

**Figure 2:**
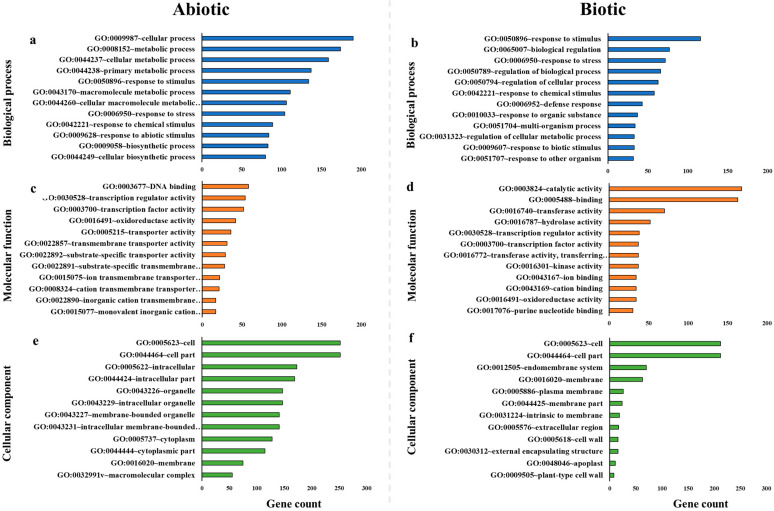
Gene ontology enrichment analysis of the DEGs under abiotic (**a**,**c**,**e**) and biotic (**b**,**d**,**f**) stresses. The enriched genes were sorted into three categories according to gene function and biological process, i.e., genes involved in molecular functions and genes responsible for synthesis and organization of cellular components.

GO enrichment analysis was performed to identify the function of the differential and common up-/downregulated DEGs in response to biotic and abiotic stresses. A total of 457 and 547 DEGs in the samples exposed to biotic and abiotic stresses were, respectively. analyzed to conduct GO analysis. GO analysis grouped the upregulated DEGs into the categories of biological process, cellular component, and molecular function. The highest frequencies of biological process-related DEGs in the samples under biotic stress belonged to the response to stimulus (26.48%), biological regulation (17.58%), and response to stress (16.44%) categories. Concurrently, cellular (37.85%) and metabolic (34.66%) processes showed the highest number of biological process-related DEGs in response to abiotic stress. In the cellular component category, most of the upregulated genes under biotic stress were related to the cell part (48.4%) and cell (48.4%) categories, while cell part (50.2%) and cell (50.2%) comprised the most significantly upregulated genes of the cellular component during abiotic stress. The large functional group of DEGs related to cellular processes highlights a rearrangement in plant metabolism in response to stress adaptation [31]. Furthermore, catalytic activity (38.36%) and binding (37.21%) revealed the most enriched numbers of molecular function group in the samples under biotic stress. On the other hand, DNA binding (11.55%), transcription regulator (10.76%), and transcription factor (10.36%) activities showed the highest number of molecular function-related DEGs in the samples under abiotic stress (Figure 2).

The significant upregulated DEGs involved in biological processes (response to stimulus (GO:0050896), biological regulation (GO:0065007), and response to stress (GO:0006950)) during biotic stress treatment were assigned to the following functions: defense response to pathogens (viruses, fungi, oomycetes, and bacteria), response to stress, protein kinase activity, cellulase and beta-glucosidase activity, MAP kinase activity, innate immune response, camalexin biosynthetic process, protein phosphorylation, calmodulin binding, induced systemic and systemic acquired resistance, signal transduction, circadian rhythm, leaf senescence, DNA-binding transcription factor activity, growth regulation, gene expression regulation, response to oxidative stress, protein ubiquitination, response to hormones, and programmed cell death. On the other hand, significant upregulated DEGs were involved in the following biological processes: cellular (GO:0009987) and metabolic (GO:0008152) processes in the abiotic stress treatment assigned to functions, including proteolysis involved in the cellular protein catabolic process, DNA-binding transcription factor activity, response to salt stress, response to desiccation, response to hormones, response to heat and cold, photosynthesis, protein ubiquitination, protein phosphorylation, heat and cold acclimation, response to water deprivation, regulation of cellular response to alkaline pH, protein binding, response to oxidative stress, chaperone binding, cellular respiration, response to osmotic stress, drought recovery, response to freezing and salt stress, protein stabilization, heat shock protein binding, proline biosynthetic process, abscisic acid biosynthetic process, signal transduction, malate dehydrogenase (decarboxylating) (NADP+) activity, lipid metabolic process, protein kinase activity, regulation of cellular protein metabolic process, lipid oxidation, circadian rhythm, and protein folding. In the cellular component, the most enriched GO in terms of the cell part and cell during biotic and abiotic stresses were signal transduction, cellular response to oxidative stress, cellular oxidant detoxification, respiratory burst, cell redox homeostasis, response to oxidative stress and reactive oxygen species, protein phosphorylation, defense response, kinase activity, cellular response to freezing, cell wall modification, pectin esterase inhibitor activity, chitinase activity, PAMP-triggered immunity signaling pathway, glutathione metabolic process, programmed cell death, lipid and carbohydrate metabolic process, pattern recognition receptor signaling pathway, proteolysis involved in cellular protein catabolic process, regulation of reactive oxygen species metabolic process, chaperone-mediated protein folding, cytochrome-c oxidase activity, mitochondrial electron transport, electron transfer activity, regulation of cellular response to alkaline pH, proline biosynthetic process, ATP synthesis-coupled electron transport, and gene silencing by RNA-directed DNA methylation. In addition, the significant upregulated DEGs involved in molecular functions, including catalytic activity (GO:0003824) and binding (GO:0005488) in the biotic stress treatment, contained activities such as a defense response to pathogens, response to stress, camalexin biosynthetic process, induced systemic resistance and systemic acquired resistance, protein binding, response to hormones, hormone-mediated signaling pathway, response to oxidative stress, protein kinase activity, protein phosphorylation, protein ubiquitination, cellulase activity, signal transduction, regulation of growth, MAP kinase activity, RNA binding, transcription regulatory region sequence-specific DNA binding, DNA-binding transcription factor activity, regulation of gene expression, RNA processing, respiratory burst, programmed cell death, production of siRNA involved in RNA interference, methylation, metalloendopeptidase activity, gene silencing by miRNA, ribonuclease III activity, innate immune response, signal transduction, glutathione transferase activity, and calmodulin binding. The most enriched DEGs for molecular functions, including DNA binding (GO:0003677), transcription regulator (GO:0030528), and transcription factor (GO:0003700) activities in the abiotic stress treatment contained functions such as response to stress, response to oxidative stress, protein ubiquitination, transcription regulatory region sequence-specific DNA binding, DNA-binding transcription factor activity, hyperosmotic salinity response, regulation of transcription and gene expression, response to hormones, response to water deprivation, heat acclimation, RNA secondary structure unwinding, RNA binding, mRNA export from the nucleus, alternative mRNA splicing, transcription coactivator activity, and the mitochondria-nucleus signaling pathway. Our findings are in agreement with other studies which showed that the large functional groups of DEGs were related to metabolic processes, regulatory function, and response to stimulus [16,31].

### 2.3. KEGG Analysis of DEGs

A total of five significant pathways, i.e., phenylpropanoid biosynthesis, plant-pathogen interaction, glutathione metabolism, amino sugar and nucleotide sugar metabolism, and tropane, piperidine, and pyridine alkaloid biosynthesis, were enriched with upregulated DEGs in the Kyoto Encyclopedia of Genes and Genomes (KEGG) analysis during biotic stresses, whereas four significant enriched pathways in response to abiotic stresses were protein processing in the endoplasmic reticulum (ER), oxidative phosphorylation, plant hormone signal transduction, and carotenoid biosynthesis (Table 1). Among the pathways characteristic of biotic stresses, phenylpropanoid and glutathione have been shown to mediate resistance to plant pathogens. Phenylpropanoids play a role in plant responses to biotic stresses and disease resistance [32,33]. An increase in phenylalanine content affects the biosynthesis of phenylpropanoid compounds, leading to increased resistance to oxidative stress and fungal infection [33]. Moreover, glutathione plays a crucial role in plant responses during biotic stresses and plant resistance to pathogens [34]. DEGs annotated to protein processing in the ER, oxidative phosphorylation, and plant hormone signal transduction were upregulated in response to abiotic stresses. The ER regulates stress responses in plants and is related to abiotic stress tolerance [35,36], mitigating stress damage and providing stress tolerance to plants [37]. ER stress signaling is involved in the response to abiotic stresses, including heat and osmotic stresses [38]. Oxidative phosphorylation produces adenosine triphosphate (ATP) required for providing the necessary energy for plant survival and development [39,40]. Cell death has been suggested to be controlled through various pathways, e.g., the perturbation of oxidative phosphorylation [41]. Phytohormones regulate a number of plant cellular processes, such as abiotic stress tolerance and plant defense response, and coordinate signal transduction pathways during an abiotic stress response. They are thereby considered potential targets for producing abiotic stress-tolerant plants [42].

### 2.4. Differential Stress-Responsive Groups

Here, we functionally grouped the highest upregulated genes in response to biotic and abiotic stresses. Our results revealed that the stress response types varied in both biotic and abiotic stresses, highlighting different stress-responsive pathways involved in plant-stress interactions. Heat shock proteins (HSPs) and heat shock transcription factors (HSFs) were among the most upregulated responsive groups under abiotic stress. The role of HSPs has previously been reported in the development of tolerance against various abiotic stresses [43], which acts through the regulation of protein folding and unfolding, subcellular localization of proteins, signal transduction, transcriptional activation and degradation of unfolded proteins, resulting in protein quality control and cellular homeostasis [43,44,45]. HSFs are also considered one of the key components of the signal transduction cascade causing the regulation and expression of genes involved in abiotic stress responses [46]. On the other hand, a number of specific stress-responsive groups were revealed during biotic stresses. Calcium (Ca) and calmodulin (CaM)-related responses were found to be upregulated in response to biotic stresses. Ca and CaM are crucial messengers in stress signaling by eliciting responses to stresses [47]. In addition, glutathione S-transferases (GSTs)-related DEGs were upregulated during biotic stresses. Our findings are in agreement with other studies that showed GST genes to be specifically induced in response to plant pathogens, which may contribute to disease resistance [48]. Some GSTs are remarkably induced in the early stages of bacterial, viral, and fungal infections. GSTs detoxify toxic substances, attenuate oxidative stress, and participate in hormone transport [48]. Furthermore, we identified induced DEGs of pathogenesis-related (PR) proteins that were responsive to phytopathogens. PR proteins play key roles in plant innate immune systems and cause enhanced resistance against biotic stresses [49] via inducing plant cell wall reinforcement, suppressing pathogen enzymes, degrading PR proteins, and inducing defense mechanisms and their direct antimicrobial activity against pathogens [50]. In addition, our findings showed that a number of membrane- and cell wall-related DEGs were induced under biotic stresses. Induced Ca-related DEGs may be responsible for the biochemical modification of the membrane and the cell wall. Ca plays a key role in maintaining the structural rigidity of cell walls and membranes [51]. Pattern recognition receptors perceive pathogen-associated molecular patterns in the plasma membrane and initiate pattern-triggered immunity (PTI) during biotic stresses [11,52,53]. PTI can be triggered by host-derived molecules from the cell wall [54]. Cell wall proteins play crucial roles in plants during stress [55]. Plasma membrane-localized receptor-like kinases and receptor-like proteins such as pattern recognition receptors play a critical role in adapting to biotic stresses [56]. In addition, plants sense the pathogens and modify their cell wall components to induce a defense response [57].

### 2.5. Identification of DEGs Encoding Transcription Factors in Response to Biotic/Abiotic Stresses

In the present study, a total of 11 and 15 TF families were significantly upregulated in response to biotic and abiotic stresses, respectively (Table S1). WRKY family showed the highest TF number (15) in response to biotic stresses, whereas AP2/EREBP family included the most presented TF (15) during abiotic stresses (Table 2). WRKY TFs are a large family of regulatory proteins and are involved in plant defense responses to biotic and abiotic stresses [58,59,60]. Bacterial, viral, and fungal infections can activate WRKY TF genes, which subsequently mediate resistance to plant pathogens [61,62,63,64,65,66]. On the other hand, AP2/EREBP, a large family of TF genes in plants [67], is involved in various abiotic stress responses, including genes related to drought [68], low temperature [69], and high salt concentrations [68]. In addition, TFs, i.e., bHLH, C2H2, C3H, CCAAT-HAP2, HSF, MYB, NAC, and WRKY were the common TFs in response to biotic and abiotic stresses. On the other hand, C2C2-CO-like, C2C2-Dof, G2-like, GRAS, Homeobox, and RAV were specifically detected in response to abiotic stresses, while TFs including REM and Trihelix were only identified against biotic stresses.

### 2.6. Protein-Protein Interaction

**Figure 3:**
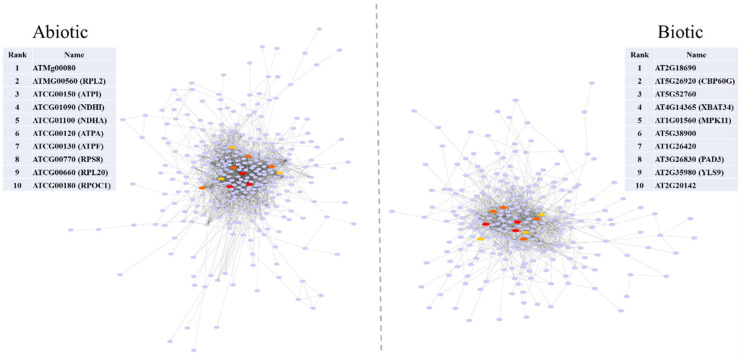
A protein-protein interaction network highlights hub genes involved in biotic and abiotic stresses in *Arabidopsis*. The most important hubs are ranked based on their importance in the network. The red, orange, and yellow nodes indicate the hub genes in each module.

In this study, we constructed a PPI network and identified the most significant hub genes. The network showed 259/333 nodes and 1283/2189 edges during biotic and abiotic stresses, respectively. The most significant hubs in the biotic stress network were transmembrane protein (AT2G18690), calmodulin-binding protein (CBP60G), copper transport protein family (AT5G52760), hypothetical protein (XBAT34), and mitogen-activated protein kinase 11 (MPK11), while a mitochondrial ribosomal protein (ATMg00080), ribosomal protein L2 (RPL2), ATP synthase CF0 A subunit (ATPI), NADH dehydrogenase subunit (NDHI), and NADH dehydrogenase subunit 1 (NDHA) represented the most significant nodes in the abiotic stress network (Figure 3).

CaM-binding proteins are involved in biotic and abiotic stress responses. Among them, CBP60g regulates SA biosynthesis induced by microbe-associated molecular patterns. The overexpression of CBP60g in *Arabidopsis* increased SA accumulation, tolerance to drought stress, expression of the defense genes, and resistance to *Pseudomonas syringae* [70]. Mitogen-activated protein kinases (MAPK) cause cellular signal transduction during stress responses. Treatment with flg22, a pathogen-associated molecular pattern, was found to increase MPK11 expression in *A. thaliana* [71]. Copper (Cu) acts as a redox-active cofactor in a number of plant proteins and is required for a wide number of critical biological processes including mitochondrial respiration, cell wall metabolism, oxidative stress protection, response to pathogens, and ethylene perception [72,73,74]. Cu is suggested to play a role in antimicrobial activity through the alteration and inhibition of protein activity, the denaturation of nucleic acids, and the modulation of plasma membrane permeabilization [75]. *Xanthomonas oryzae* has been shown to overcome rice defenses by regulating host Cu redistribution [74].

Mitochondrial F(1)F(0)-ATPase, an important enzyme in plant metabolism, provides ATP for cells. The overexpression of a mitochondrial ATP synthase small subunit gene reportedly confers resistance to several abiotic stresses (salts, drought, oxidative and cold stresses) in *A. thaliana,* suggesting F(1)F(0)-ATPase to play a role in stress tolerance [76]. ATP synthase subunits (e.g., ATPase F0) and NADH dehydrogenase were also found to change in plants under drought stress [77,78], and these changes may result in an increase of energy production in response to water deficit to inhibit stress damage [79]. NADH dehydrogenase enhances tolerance to environmental stresses, including drought and elevated light, and is thought to prevent cellular damage [80].

### 2.7. Identification of miRNAs Targeting Downregulated Genes

miRNAs are an extensive class of non-coding RNAs that play a key role in the regulation of various biological processes through post-transcriptional regulation of gene expression. In this study, we identified 84 miRNAs belonging to 33 miRNA families (Table S2), a high proportion of which are involved in various biological processes.

The analysis of miRNAs during biotic and abiotic stresses exhibited 5 and 25 miRNA families, respectively, showing their role in the downregulation of *A. thaliana* genes in response to stresses.

ath-miR172 was predicted to have the highest frequency among downregulated gene targets, with eight targets during biotic stresses, whereas ath-miR858 and ath-miR5665 were identified with the highest targets (eight and six targets) during abiotic stresses, significantly showing the highest number of miRNAs targeting downregulated genes. In addition, ath-miR414 and ath-miR5658 were identified to be commonly expressed in response to both biotic and abiotic stresses. Similarly, miR172 was found to significantly downregulate a number of gene targets during viral infections in *A. thaliana* [81]. miR172 also reportedly regulates genes during biotic and abiotic stresses [82,83]. MiR858 has been shown to target regulatory factors, resulting in the regulation of a number of plant functions, including hormonal and stress responses [84]. In addition, the role of miR858 has been established in drought stress response [85], and this miRNA was upregulated in response to salt stress [86]. Moreover, two common microRNAs (miR414, miR5658) detected in response to biotic and abiotic stresses were the key regulators of salt-responsive genes [87]. MiR5658 plays prominent roles in the regulation of processes involved in responses to biotic and abiotic stresses [88,89,90]. Our results may shed light on miRNA functions in plant responses to biotic and abiotic stresses.

## 3. Materials and Methods

### 3.1. Data Collection and Preprocessing

**Figure 4:**
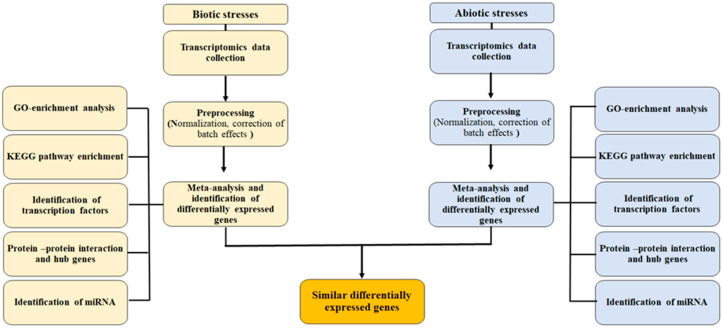
Meta-Analysis of an Expression Dataset.

The raw expression data (RNA-seq) for biotic and abiotic responses were obtained from the ArrayExpress of the European Bioinformatics Institute (http://www.ebi.ac.uk/arrayexpress; 15 September 2020). The data sets were filtered for *Arabidopsis thaliana,* to only include RNA-seq data. The search yielded 12 entries in 11 papers, as detailed in Table 3. After trimming the adaptor sequences and low-quality reads based on their Phred quality scores [91], we mapped the checked reads onto the *Arabidopsis* reference genome. Expression profiling analysis was carried out using CLC Genomic Workbench version 10 (CLC Bio, Qiagen). The raw expression data pertaining to each data set were normalized with counts per million (CPM). The workflow is presented in Figure 4.

### 3.2. Meta-Analysis of an Expression Dataset

We conducted a meta-analysis independently on an integrated dataset pertaining to DEGs in biotic/abiotic stresses. Each dataset was grouped into a stress class and a healthy class according to the type of stress involved. Before the meta-analysis, the SVA R package was applied to correct for the batch effect, according to the empirical Bayes method [103]. Fisher’s method was used for detecting DEGs (FDR < 0.01) [104]. The log ratio of means (ROM) was used to measure gene expression level [105]. ROM was calculated using the following equation:

(1) $$y_{gn}=ln\left[\frac{{\bar{r}}_{gr}}{{\bar{r}}_{gs}}\right]$$

where *y_gn_*, *r_gr_*, and *r_gs_* represent ROM and mean expression levels of the stress and healthy classes for each gene in the dataset, respectively. Data were preprocessed and analyzed using Bioconductor packages (http://www.bioconductor.org; 15 September 2020), including MetaMA.

### 3.3. Gene Enrichment Analysis and Functional Analysis

The genes were analyzed after being selected by the meta-analysis. In the first instance, the co-occurrence of DEG with the resulting list was represented with Venn’s diagrams using Venny 2.0 (http://bioinfogp.cnb.csic.es/tools/venny/; 15 September 2020). Enrichment analysis of Gene Ontology (GO) was performed on significant DEGs, which had been obtained from the meta-analysis. The enrichment analysis was conducted using the AgriGO platform [106]. Information on gene ontology was extracted based on GO terms for biological processes, cellular components, and molecular functions when a significant threshold of FDR < 0.05 was obtained. The pathway analysis of the Kyoto Encyclopedia of Genes and Genomes (KEGG) was used to elucidate the enriched pathways of the DEGs (http://david.abcc.ncifcrf.gov/; 15 September 2020). To identify transcription factors among the DEGs, a list of *Arabidopsis* transcription factors was obtained from the AGRIS database (https://agris-knowledgebase.org/AtTFDB/; 15 September 2020).

### 3.4. Protein-Protein Interactions and Network Construction

The network analysis of protein-protein interactions (PPI) was performed to uncover any plausible interactions among proteins with significantly different DEGs. The STRING database was employed to enable the PPI network analysis [107], and Cytoscape software was used for visualizing the interaction networks.

### 3.5. Prediction of Potential miRNAs

The identification of miRNAs associated with biotic and abiotic stresses is necessary for understanding how plants respond defensively to stresses. Identifying potential miRNAs is possible with the psRNATarget server (http://plantgrn.noble.org/psRNATarget/; 15 September 2020), wherein we set the parameters to default, except for maximum expectation, which was set to 3 in this study.

## 4. Conclusions

This study provides valuable information on *A. thaliana* in terms of differential and common host responses against biotic and abiotic stresses. As plants are simultaneously exposed to both biotic and abiotic stresses in the field and in natural ecosystems, the information generated here will help us identify common and specific genes and pathways during biotic and abiotic stresses. The common and specific genes, as potential candidate targets, may provide new insights for developing stress-resistant breeding and the genetic manipulation of crop plants via integrating biotic and abiotic resistance traits. Defense-related pathways will be determined based on this, and they may simultaneously develop plant resistance to biotic and abiotic stresses and improve crop productivity. Further studies are required to elucidate the molecular mechanisms and validate the function of responsive DEGs, TFs, and miRNAs that regulate plant responses to biotic and abiotic stresses.

## Figures and Tables

**Table 1:** KEGG pathway enrichment of the total differentially expressed genes (DEGs) in response to biotic and abiotic stresses.

Stress	Pathway	Gene Count	Adjusted *p* Value
Biotic	Plant-pathogen interaction	11	0.000170
	Phenylpropanoid biosynthesis	11	0.000026
	Glutathione metabolism	9	0.000021
	Amino sugar and nucleotide sugar metabolism	6	0.029237
Abiotic	Protein processing in the endoplasmic reticulum	16	0.000002
	Plant hormone signal transduction	12	0.003789
	Oxidative phosphorylation	7	0.049932
	Carotenoid biosynthesis	4	0.011602

**Table 2:** Identified transcription factors (TF) regulated in response to biotic and abiotic stresses.

TF Family	No. of TF in Abiotic	No. of TF in Biotic
AP2-EREBP	15	2
bHLH	2	1
C2C2-CO-like	1	-
C2C2-Dof	2	-
C2H2	8	4
C3H	3	1
CCAAT-HAP2	1	1
G2-like	1	-
GRAS	1	-
Homeobox	2	-
HSF	5	1
MYB	2	3
NAC	6	6
RAV	1	-
REM	-	1
Trihelix	-	1
WRKY	1	15

**Table 3:** Transcriptomic raw data related to different biotic and abiotic stress studies of *Arabidopsis thaliana* used for the current meta-analysis.

Accession Number	Stress	Sample Number	Control Number	Related Article
E-MTAB-4151	*Pseudomonas syringae* pv. *maculicola*	24	12	[92]
E-GEOD-53641	*Hyaloperonospora arabidopsidis*	204	72	[93]
E-GEOD-34241	*Fusarium oxysporum*	8	4	[94]
E-MTAB-4416	*Pseudomonas syringae*	6	3	[95]
E-GEOD-56922	*Cabbage leaf curl virus*	8	4	[96]
E-MTAB-4281	*Botrytis cinerea*	4	2	[97]
E-MTAB-4450	*Pseudomonas syringae*	18	6	[98]
E-MTAB-3908	Drought	21	11	EBI
E-MTAB-5009	Mild drought	17	9	[99]
E-GEOD-63406	Temperature	9	3	[100]
PRJNA240248	Salt	4	2	[101]
PRJNA295091	Salt and heat stress	12	3	[102]

EBI—The European Bioinformatics Institute.

## Data Availability

Not applicable.

## Supplementary Materials

The following supporting information can be downloaded at: https://www.mdpi.com/article/10.3390/plants11040502/s1, Table S1: List of the identified DEGs by the meta-analysis, Table S2: Gene ontology enrichment analysis of DEGs under abiotic and biotic stresses, Table S3: Identified transcription factors in response to biotic and abiotic stresses, Table S4: miRNAs associated with the DEGs were discovered using the computational algorithm psRNATarget server.

## References

[1] Bhar A., Chakraborty A., Roy A. (2022). Plant responses to biotic stress: Old memories matter. Plants.

[2] Yang L., Wang Z., Hua J. (2021). A meta-analysis reveals opposite effects of biotic and abiotic stresses on transcript levels of *Arabidopsis* intracellular immune receptor genes. Front. Plant Sci..

[3] Costa-Mattioli M., Walter P. (2020). The integrated stress response: From mechanism to disease. Science.

[4] Bai Y., Kissoudis C., Yan Z., Visser R.G., van der Linden G. (2018). Plant behaviour under combined stress: Tomato responses to combined salinity and pathogen stress. Plant J..

[5] Saijo Y., Loo E.P.I. (2020). Plant immunity in signal integration between biotic and abiotic stress responses. New Phytol..

[6] Atkinson N.J., Urwin P.E. (2012). The interaction of plant biotic and abiotic stresses: From genes to the field. J. Exp. Bot..

[7] Nejat N., Mantri N. (2017). Plant immune system: Crosstalk between responses to biotic and abiotic stresses the missing link in understanding plant defence. Curr. Issues Mol. Biol..

[8] Gimenez E., Salinas M., Manzano-Agugliaro F. (2018). Worldwide research on plant defense against biotic stresses as improvement for sustainable agriculture. Sustainability.

[9] Zhang N., Zhou S., Yang D., Fan Z. (2020). Revealing shared and distinct genes responding to JA and SA signaling in *Arabidopsis* by meta-analysis. Front. Plant Sci..

[10] Jiang Z., He F., Zhang Z. (2017). Large-scale transcriptome analysis reveals *Arabidopsis* metabolic pathways are frequently influenced by different pathogens. Plant Mol. Biol..

[11] Jones J.D.G., Dangl J.L. (2016). The plant immune system. Nature.

[12] Huot B., Yao J., Montgomery B.L., He S.Y. (2014). Growth–defense tradeoffs in plants: A balancing act to optimize fitness. Mol. Plant.

[13] Kissoudis C., Sunarti S., van de Wiel C., Visser R.G., van der Linden C.G., Bai Y. (2016). Responses to combined abiotic and biotic stress in tomato are governed by stress intensity and resistance mechanism. J. Exp. Bot..

[14] Kapoor D., Bhardwaj S., Landi M., Sharma A., Ramakrishnan M., Sharma A. (2020). The impact of drought in plant metabolism: How to exploit tolerance mechanisms to increase crop production. Appl. Sci..

[15] Tahmasebi A., Ashrafi-Dehkordi E., Shahriari A.G., Mazloomi S.M., Ebrahimie E. (2019). Integrative meta-analysis of transcriptomic responses to abiotic stress in cotton. Prog. Biophys. Mol. Biol..

[16] Ashrafi-Dehkordi E., Alemzadeh A., Tanaka N., Razi H. (2018). Meta-analysis of transcriptomic responses to biotic and abiotic stress in tomato. PeerJ.

[17] Bolton M.D. (2009). Primary metabolism and plant defense—Fuel for the fire. Mol. Plant-Microbe Interact..

[18] Rojas C.M., Senthil-Kumar M., Tzin V., Mysore K. (2014). Regulation of primary plant metabolism during plant-pathogen interactions and its contribution to plant defense. Front. Plant Sci..

[19] Mittler R. (2006). Abiotic stress, the field environment and stress combination. Trends Plant Sci..

[20] Bilgin D.D., Zavala J.A., Zhu J.I.N., Clough S.J., Ort D.R., Delucia E.H. (2010). Biotic stress globally downregulates photosynthesis genes. Plant Cell Environ..

[21] Dong Y.J., Wang Z.L., Zhang J.W., Liu S., He Z.L., He M.R. (2015). Interaction effects of nitric oxideand salicylic acid in alleviating salt stress of *Gossypium hirsutum* L. J. Soil Sci. Plant Nutr..

[22] Zhao F., Zhang D., Zhao Y., Wang W., Yang H., Tai F., Li C., Hu X. (2016). The difference of physiological and proteomic changes in maize leaves adaptation to drought, heat, and combined both stresses. Front. Plant Sci..

[23] Jun X.U., Wang X.Y., Guo W.Z. (2015). The cytochrome P450 superfamily: Key players in plant development and defense. J. Integr. Agric..

[24] Pandian B.A., Sathishraj R., Djanaguiraman M., Prasad P.V., Jugulam M. (2020). Role of cytochrome P450 enzymes in plant stress response. Antioxidants.

[25] Schuler M.A. (1996). The role of cytochrome P450 monooxygenases in plant-insect interactions. Plant Physiol..

[26] Li X., Zhang J.B., Song B., Li H.P., Xu H.Q., Qu B., Dang F.J., Liao Y.C. (2010). Resistance to Fusarium head blight and seedling blight in wheat is associated with activation of a cytochrome P450 gene. Phytopathology.

[27] Mazarei M., Lennon K.A., Puthoff D.P., Rodermel S.R., Baum T.J. (2003). Expression of an *Arabidopsis* phosphoglycerate mutase homologue is localized to apical meristems, regulated by hormones, and induced by sedentary plant-parasitic nematodes. Plant Mol. Biol..

[28] Rozenzvieg D., Elmaci C., Samach A., Lurie S., Porat R. (2004). Isolation of four heat shock protein cDNAs from grapefruit peel tissue and characterization of their expression in response to heat and chilling temperature stresses. Physiol. Plant..

[29] Ma W., Zhao T., Li J., Liu B., Fang L., Hu Y., Zhang T. (2016). Identification and characterization of the GhHsp20 gene family in *Gossypium hirsutum*. Sci. Rep..

[30] Cheung M.Y., Li M.W., Yung Y.L., Wen C.Q., Lam H.M. (2013). The unconventional P-loop NTPase OsYchF1 and its regulator OsGAP1 play opposite roles in salinity stress tolerance. Plant Cell Environ..

[31] Shaar-Moshe L., Hübner S., Peleg Z. (2015). Identification of conserved drought-adaptive genes using a cross-species meta-analysis approach. BMC Plant Biol..

[32] La Camera S., Gouzerh G., Dhondt S., Hoffmann L., Fritig B., Legrand M., Heitz T. (2004). Metabolic reprogramming in plant innate immunity: The contributions of phenylpropanoid and oxylipin pathways. Immunol. Rev..

[33] Geng D., Shen X., Xie Y., Yang Y., Bian R., Gao Y., Li P., Sun L., Feng H., Ma F. (2020). Regulation of phenylpropanoid biosynthesis by MdMYB88 and MdMYB124 contributes to pathogen and drought resistance in apple. Hortic. Res..

[34] Dubreuil-Maurizi C., Poinssot B. (2012). Role of glutathione in plant signaling under biotic stress. Plant Signal. Behav..

[35] Ellgaard L., Helenius A. (2003). Quality control in the endoplasmic reticulum. Nat. Rev. Mol. Cell Biol..

[36] Liu L., Li J. (2019). Communications between the endoplasmic reticulum and other organelles during abiotic stress response in plants. Front. Plant Sci..

[37] Howell S.H. (2013). Endoplasmic reticulum stress responses in plants. Annu. Rev. Plant Biol..

[38] Park C.J., Park J.M. (2019). Endoplasmic reticulum plays a critical role in integrating signals generated by both biotic and abiotic stress in plants. Front. Plant Sci..

[39] Schwarzländer M., Finkemeier I. (2013). Mitochondrial energy and redox signaling in plants. Antioxid. Redox Signal..

[40] Farhat N., Hichri S., Hildebrandt T.M., Debez A., Braun H.P. (2019). Composition and stability of the oxidative phosphorylation system in the halophile plant Cakile maritima. Front. Plant Sci..

[41] Green D.R., Reed J.C. (1998). Mitochondria and apoptosis. Science.

[42] Wani S.H., Kumar V., Shriram V., Sah S.K. (2016). Phytohormones and their metabolic engineering for abiotic stress tolerance in crop plants. Crop J..

[43] Mishra D., Shekhar S., Singh D., Chakraborty S., Chakraborty N. (2018). Heat Shock Proteins and Abiotic Stress Tolerance in Plants. Regulation of Heat Shock Protein Responses.

[44] Wang W., Vinocur B., Shoseyov O., Altman A. (2004). Role of plant heat-shock proteins and molecular chaperones in the abiotic stress response. Trends Plant Sci..

[45] Singh R.K., Jaishankar J., Muthamilarasan M., Shweta S., Dangi A., Prasad M. (2016). Genome-wide analysis of heat shock proteins in C4 model, foxtail millet identifies potential candidates for crop improvement under abiotic stress. Sci. Rep..

[46] Guo M., Liu J.H., Ma X., Luo D.X., Gong Z.H., Lu M.H. (2016). The plant heat stress transcription factors (HSFs): Structure, regulation, and function in response to abiotic stresses. Front. Plant Sci..

[47] Reddy A.S., Ali G.S., Celesnik H., Day I.S. (2011). Coping with stresses: Roles of calcium and calcium/calmodulin-regulated gene expression. Plant Cell.

[48] Gullner G., Komives T., Király L., Schröder P. (2018). Glutathione S-transferase enzymes in plant-pathogen interactions. Front. Plant Sci..

[49] Ali S., Ganai B.A., Kamili A.N., Bhat A.A., Mir Z.A., Bhat J.A., Tyagi A., Islam S.T., Mushtaq M., Yadav P. (2018). Pathogenesis-related proteins and peptides as promising tools for engineering plants with multiple stress tolerance. Microbiol. Res..

[50] Sudisha J., Sharathchandra R.G., Amruthesh K.N., Kumar A., Shetty H.S. (2012). Pathogenesis Related Proteins in Plant Defense Response. Plant Defence: Biological Control.

[51] Hepler P.K. (2005). Calcium: A central regulator of plant growth and development. Plant Cell.

[52] Gust A.A., Pruitt R., Nurnberger T. (2017). Sensing danger: Key to activating plant immunity. Trends Plant Sci..

[53] Tang D., Wang G., Zhou J.M. (2017). Receptor kinases in plant–pathogen interactions: More than pattern recognition. Plant Cell.

[54] Hamann T. (2015). The plant cell wall integrity maintenance mechanismconcepts for organization and mode of action. Plant Cell Physiol..

[55] Durufle H., San Clemente H., Balliau T., Zivy M., Dunand C., Jamet E. (2017). Cell wall proteome analysis of *Arabidopsis thaliana* mature stems. Proteomics.

[56] Burkart R.C., Stahl Y. (2017). Dynamic complexity: Plant receptor complexes at the plasma membrane. Curr. Opin. Plant Biol..

[57] Blümke A., Sode B., Ellinger D., Voigt C.A. (2015). Reduced susceptibility to Fusarium head blight in *Brachypodium distachyon* through priming with the Fusarium mycotoxin deoxynivalenol. Mol. Plant Pathol..

[58] Eulgem T., Somssich I.E. (2017). Networks of WRKY transcription factors in defense signaling. Curr. Opin. Plant Biol..

[59] Jiang Y., Duan Y., Yin J., Ye S., Zhu J., Zhang F., Lu W., Fan D., Luo K. (2014). Genome-wide identification and characterization of the Populus WRKY transcription factor family and analysis of their expression in response to biotic and abiotic stresses. J. Exp. Bot..

[60] Rushton P.J., Somssich I.E., Ringler P., Shen Q.J. (2010). WRKY transcription factors. Trends Plant Sci..

[61] Deslandes L., Olivier J., Theulières F., Hirsch J., Feng D.X., Bittner-Eddy P., Beynon J., Marco Y. (2002). Resistance to *Ralstonia solanacearum* in *Arabidopsis thaliana* is conferred by the recessive RRS1-R gene, a member of a novel family of resistance genes. Proc. Natl. Acad. Sci. USA.

[62] Knoth C., Ringler J., Dangl J.L., Eulgem T. (2007). *Arabidopsis* WRKY70 is required for full RPP4-mediated disease resistance and basal defense against *Hyaloperonospora parasitica*. Mol. Plant-Microbe Interact..

[63] Murray S.L., Ingle R.A., Petersen L.N., Denby K.J. (2007). Basal resistance against *Pseudomonas syringae* in *Arabidopsis* involves WRKY53 and a protein with homology to a nematode resistance protein. Mol. Plant Microbe Interact..

[64] Mzid R., Marchive C., Blancard D., Deluc L., Barrieu F., Corio-Costet M.F., Drira N., Hamdi S., Lauvergeat V. (2007). Overexpression of VvWRKY2 in tobacco enhances broad resistance to necrotrophic fungal pathogens. Physiol. Plant..

[65] Chen L., Zhang L., Li D., Wang F., Yu D. (2013). WRKY8 transcription factor functions in the TMV-cg defense response by mediating both abscisic acid and ethylene signaling in *Arabidopsis*. Proc. Natl. Acad. Sci. USA.

[66] Zou L., Yang F., Ma Y., Wu Q., Yi K., Zhang D. (2019). Transcription factor WRKY30 mediates resistance to Cucumber mosaic virus in *Arabidopsis*. Biochem. Biophys. Res. Commun..

[67] Sharoni A.M., Nuruzzaman M., Satoh K., Shimizu T., Kondoh H., Sasaya T., Choi I.R., Omura T., Kikuchi S. (2011). Gene structures, classification and expression models of the AP2/EREBP transcription factor family in rice. Plant Cell Physiol..

[68] Dubouzet J.G., Sakuma Y., Ito Y., Kasuga M., Dubouzet E.G., Miura S., Seki M., Shinozaki K., Yamaguchi-Shinozaki K. (2003). OsDREB genes in rice, Oryza sativa L., encode transcription activators that function in drought-, high-salt- and cold- responsive gene expression. Plant J..

[69] Qin Q.L., Liu J.G., Zhang Z., Peng R.H., Xiong A.S., Yao Q.H., Chen J.M. (2007). Isolation, optimization, and functional analysis of the cDNA encoding transcription factor OsDREB1B in *Oryza sativa* L. Mol. Breed..

[70] Wan D., Li R., Zou B., Zhang X., Cong J., Wang R., Xia Y., Li G. (2012). Calmodulin-binding protein CBP60g is a positive regulator of both disease resistance and drought tolerance in *Arabidopsis*. Plant Cell Rep..

[71] Bethke G., Pecher P., Eschen-Lippold L., Tsuda K., Katagiri F., Glazebrook J., Scheel D., Lee J. (2012). Activation of the *Arabidopsis thaliana* mitogen-activated protein kinase MPK11 by the flagellin-derived elicitor peptide, flg22. Mol. Plant-Microbe Interact..

[72] Puig S., Andrés-Colás N., García-Molina A., Penarrubia L. (2007). Copper and iron homeostasis in *Arabidopsis*: Responses to metal deficiencies, interactions and biotechnological applications. Plant Cell Environ..

[73] Burkhead J.L., Gogolin Reynolds K.A., Abdel-Ghany S.E., Cohu C.M., Pilon M. (2009). Copper homeostasis. New Phytol..

[74] Yuan M., Chu Z., Li X., Xu C., Wang S. (2010). The bacterial pathogen *Xanthomonas oryzae* overcomes rice defenses by regulating host copper redistribution. Plant Cell.

[75] Borkow G., Gabbay J. (2004). Putting copper into action: Copper-impregnated products with potent biocidal activities. FASEB J..

[76] Zhang X., Liu S., Takano T. (2008). Overexpression of a mitochondrial ATP synthase small subunit gene (AtMtATP6) confers tolerance to several abiotic stresses in Saccharomyces cerevisiae and *Arabidopsis thaliana*. Biotechnol. Lett..

[77] Plomion C., Lalanne C., Claverol S., Meddour H., Kohler A., Bogeat-Triboulot M.B., Barre A., le Provost G., Dumazet H., Jacob D. (2006). Mapping the proteome of poplar and application to the discovery of drought-stress responsive proteins. Proteomics.

[78] Koh J., Chen G., Yoo M.J., Zhu N., Dufresne D., Erickson J.E., Shao H., Chen S. (2015). Comparative proteomic analysis of *Brassica napus* in response to drought stress. J. Proteome Res..

[79] Wang X., Cai X., Xu C., Wang Q., Dai S. (2016). Drought-responsive mechanisms in plant leaves revealed by proteomics. Int. J. Mol. Sci..

[80] Sweetman C., Waterman C.D., Rainbird B.M., Smith P.M., Jenkins C.D., Day D.A., Soole K.L. (2019). AtNDB2 is the main external NADH dehydrogenase in mitochondria and is important for tolerance to environmental stress. Plant Physiol..

[81] Tahmasebi A., Khahani B., Tavakol E., Afsharifar A., Shahid M.S. (2021). Microarray analysis of *Arabidopsis thaliana* exposed to single and mixed infections with Cucumber mosaic virus and turnip viruses. Physiol. Mol. Biol. Plants.

[82] Liu H.H., Tian X., Li Y.J., Wu C.A., Zheng C.C. (2008). Microarray-based analysis of stress-regulated microRNAs in *Arabidopsis thaliana*. RNA.

[83] Sunkar R., Li Y.F., Jagadeeswaran G. (2012). Functions of microRNAs in plant stress responses. Trends Plant Sci..

[84] Sharma D., Tiwari M., Pandey A., Bhatia C., Sharma A., Trivedi P.K. (2016). MicroRNA858 is a potential regulator of phenylpropanoid pathway and plant development. Plant Physiol..

[85] Gao F., Wang N., Li H., Liu J., Fu C., Xiao Z., Wei C., Lu X., Feng J., Zhou Y. (2016). Identification of drought-responsive microRNAs and their targets in *Ammopiptanthus mongolicus* by using high-throughput sequencing. Sci. Rep..

[86] Yang Z., Zhu P., Kang H., Liu L., Cao Q., Sun J., Dong T., Zhu M., Li Z., Xu T. (2020). High-throughput deep sequencing reveals the important role that microRNAs play in the salt response in sweet potato (*Ipomoea batatas* L.). BMC Genom..

[87] Wang J., Xu M., Li Z., Ye Y., Rong H., Xu L.A. (2018). Tamarix microRNA profiling reveals new insight into salt tolerance. Forests.

[88] Barozai M.Y.K., Kakar S., Sarangzai A.M. (2013). Profiling the carrot (*Daucus carota* L.) microRNAs and their targets. Pak. J. Bot..

[89] Wen C.L., Cheng Q., Zhao L.Q., Mao A.J., Yang J.J., Yu S.C., Weng Y., Xu Y. (2016). Identification and characterisation of Dof transcription factors in the cucumber genome. Sci. Rep..

[90] Liang C., Liu H., Hao J., Li J., Luo L. (2019). Expression profiling and regulatory network of cucumber microRNAs and their putative target genes in response to cucumber green mottle mosaic virus infection. Arch. Virol..

[91] Ewing B., Green P. (1998). Base-calling of automated sequencer traces using phred. II. Error probabilities. Genome Res..

[92] Bernsdorff F., Döring A.C., Gruner K., Schuck S., Bräutigam A., Zeier J. (2016). Pipecolic acid orchestrates plant systemic acquired resistance and defense priming via salicylic acid-dependent and-independent pathways. Plant Cell.

[93] Asai S., Rallapalli G., Piquerez S.J., Caillaud M.C., Furzer O.J., Ishaque N., Wirthmueller L., Fabro G., Shirasu K., Jones J.D. (2014). Expression profiling during *Arabidopsis*/downy mildew interaction reveals a highly-expressed effector that attenuates responses to salicylic acid. PLoS Pathog..

[94] Zhu Q.H., Stephen S., Kazan K., Jin G., Fan L., Taylor J., Dennis E.S., Helliwell C.A., Wang M.B. (2013). Characterization of the defense transcriptome responsive to *Fusarium oxysporum*-infection in *Arabidopsis* using RNA-seq. Gene.

[95] Filichkin S.A., Cumbie J.S., Dharmawardhana P., Jaiswal P., Chang J.H., Palusa S.G., Refy A.S.N., Megraw M., Mockler T.C. (2015). Environmental stresses modulate abundance and timing of alternatively spliced circadian transcripts in *Arabidopsis*. Mol. Plant.

[96] Zorzatto C., Machado J.P.B. (2015). Lopes, K.V.; Nascimento, K.J.; Pereira, W.A.; Brustolini, O.J.; Reis, P.A.B.; Calil, I.P.; Deguchi, M.; Sachetto-Martins, G.; et al. NIK1-mediated translation suppression functions as a plant antiviral immunity mechanism. Nature.

[97] Lai Z., Schluttenhofer C.M., Bhide K., Shreve J., Thimmapuram J., Lee S.Y., Jun D.-Y., Mengiste T. (2014). MED18 interaction with distinct transcription factors regulates multiple plant functions. Nat. Commun..

[98] Howard B.E., Hu Q., Babaoglu A.C., Chandra M., Borghi M., Tan X., He L., Winter-Sederoff H., Gassmann W., Heber S. (2013). High-throughput RNA sequencing of pseudomonas-infected *Arabidopsis* reveals hidden transcriptome complexity and novel splice variants. PLoS ONE.

[99] Clauw P., Coppens F., Korte A., Herman D., Slabbinck B., Dhondt S., van Daele T., de Milde L., Vermeersch M., Inzé D. (2016). Leaf growth response to mild drought: Natural variation in *Arabidopsis* sheds light on trait architecture. Plant Cell.

[100] Schlaen R.G., Mancini E., Sanchez S.E., Perez-Santángelo S., Rugnone M.L., Simpson C.G., Brown J.W.S., Zhang X., Chernomoretz A., Yanovsky M.J. (2015). The spliceosome assembly factor GEMIN2 attenuates the effects of temperature on alternative splicing and circadian rhythms. Proc. Natl. Acad. Sci. USA.

[101] Feng J., Li J., Gao Z., Lu Y., Yu J., Zheng Q., Yan S., Zhang W., He H., Zhu Z. (2015). SKIP confers osmotic tolerance during salt stress by controlling alternative gene splicing in *Arabidopsis*. Mol. Plant.

[102] Suzuki N., Bassil E., Hamilton J.S., Inupakutika M.A., Zandalinas S.I., Tripathy D., Luo Y., Dion E., Fukui G., Mittler R. (2016). ABA is required for plant acclimation to a combination of salt and heat stress. PLoS ONE.

[103] Leek J.T., Johnson W.E., Parker H.S., Jaffe A.E., Storey J.D. (2012). The sva package for removing batch effects and other unwanted variation in high-throughput experiments. Bioinformatics.

[104] Benjamini Y., Hochberg Y. (1995). Controlling the false discovery rate: A practical and powerful approach to multiple testing. J. R. Stat. Soc. Ser. B.

[105] Hu P., Greenwood C.M., Beyene J. (2009). Using the ratio of means as the effect size measure in combining results of microarray experiments. BMC Syst. Biol..

[106] Du Z., Zhou X., Ling Y., Zhang Z., Su Z. (2010). agriGO: A GO analysis toolkit for the agricultural community. Nucleic Acids Res..

[107] Szklarczyk D., Franceschini A., Wyder S., Forslund K., Heller D., Huerta-Cepas J., Simonovic M., Roth A., Santos A., von Mering C. (2015). STRING v10: Protein-protein interaction networks, integrated over the tree of life. Nucleic Acids Res..

