# Supplementary material for: Meta-Analysis of Common and Differential Transcriptomic Responses to Biotic and Abiotic Stresses in Arabidopsis thaliana
Source: Plants (Basel). 2022 Feb 12;11(4):502. doi: 10.3390/plants11040502 (PMC8877356; doi:10.3390/plants11040502)
Supplement: Supplementary file 1 [file plants-11-00502-s001.zip › plants-1584261-supplementary.pdf]

plants

MDPI

Supplementary Material

Table S1. List of the identified DEGs by meta- analysis.

Stress	ID	FC	*p*-value	
Biotic	AT2G44890	3.959559	0.000104	Up
Biotic	AT1G09935	3.919835	0.000715	Up
Biotic	AT4G34470	3.562859	0.007997	Up
Biotic	AT5G37640	3.203562	0.006749	Up
Biotic	AT3G14450	2.99951	0.001327	Up
Biotic	AT2G30770	2.918921	0.000225	Up
Biotic	AT3G12230	2.912123	0.000268	Up
Biotic	AT1G64160	2.88458	0.003007	Up
Biotic	AT1G33950	2.872597	0.002679	Up
Biotic	AT5G56510	2.857949	0.007553	Up
Biotic	AT5G45090	2.825806	0.000257	Up
Biotic	AT5G36970	2.813229	8.13E-05	Up
Biotic	AT3G03670	2.81107	0.00051	Up
Biotic	AT5G06740	2.800048	0.000325	Up
Biotic	AT4G11070	2.763891	0.003922	Up
Biotic	AT2G24285	2.761538	0.001242	Up
Biotic	AT5G39120	2.650048	0.008231	Up
Biotic	AT5G22560	2.647134	0.007283	Up
Biotic	AT2G31335	2.63938	0.000902	Up
Biotic	AT2G21900	2.630096	0.008572	Up
Biotic	AT1G06135	2.626024	8.34E-06	Up
Biotic	AT3G13610	2.622067	2.04E-05	Up
Biotic	AT1G05880	2.620229	0.00188	Up
Biotic	AT5G25260	2.616393	0.003521	Up
Biotic	AT5G46960	2.584948	0.00011	Up
Biotic	AT2G29470	2.571698	0.000553	Up
Biotic	AT5G40990	2.566352	9.75E-05	Up
Biotic	AT4G19925	2.531731	0.006113	Up
Biotic	AT2G45760	2.528714	0.002692	Up
Biotic	AT1G75830	2.507154	3.14E-05	Up
Biotic	AT3G25490	2.505425	0.007984	Up
Biotic	AT2G29150	2.486676	0.003318	Up
Biotic	AT5G64790	2.46628	0.006858	Up
Biotic	AT3G46080	2.460804	0.006172	Up
Biotic	AT3G21520	2.45262	7.09E-05	Up
Biotic	AT5G23160	2.450968	0.000222	Up
Biotic	AT1G15520	2.448128	1.8E-05	Up
Biotic	AT4G11655	2.445632	0.00074	Up
Biotic	AT5G38344	2.424099	0.000501	Up
Biotic	AT1G71390	2.419528	8.03E-05	Up
Biotic	AT1G19250	2.414245	5.03E-05	Up
Biotic	AT5G19880	2.398417	0.000928	Up
Biotic	AT1G13310	2.3956	0.004868	Up
Biotic	AT2G35965	2.394258	0.001587	Up

	Biotic	AT2G23830	2.391475	0.000303	Up
	Biotic	AT1G66600	2.39027	1.34E-05	Up
	Biotic	AT1G76470	2.349222	0.000513	Up
	Biotic	AT1G44130	2.344455	5.32E-05	Up
	Biotic	AT1G33960	2.316374	0.007516	Up
	Biotic	AT1G53620	2.316024	0.002174	Up
	Biotic	AT1G13340	2.305093	0.000148	Up
	Biotic	AT1G02580	2.299597	0.000929	Up
	Biotic	AT2G43570	2.279709	3.9E-05	Up
	Biotic	AT1G32350	2.264254	0.000499	Up
	Biotic	AT1G57630	2.252161	2.87E-05	Up
	Biotic	AT1G26410	2.244849	3.8E-07	Up
	Biotic	AT4G37710	2.226852	0.000141	Up
	Biotic	AT5G11210	2.22462	5.96E-06	Up
	Biotic	AT5G13320	2.21927	0.004205	Up
	Biotic	AT5G38900	2.217096	1.61E-05	Up
	Biotic	AT3G13433	2.196928	0.000385	Up
	Biotic	AT5G24540	2.186102	0.000154	Up
	Biotic	AT1G43910	2.176123	3.16E-05	Up
	Biotic	AT4G21840	2.170849	1.34E-05	Up
	Biotic	AT4G33020	2.166246	0.001905	Up
	Biotic	AT1G66700	2.162867	6.47E-06	Up
	Biotic	AT2G29350	2.154972	1.26E-05	Up
	Biotic	AT5G38350	2.153733	0.000596	Up
	Biotic	AT4G19970	2.150172	5.31E-05	Up
	Biotic	AT3G11340	2.129298	0.000259	Up
	Biotic	AT5G64810	2.125814	1.81E-06	Up
	Biotic	AT3G53150	2.119596	0.001403	Up
	Biotic	AT3G45330	2.106738	0.000216	Up
	Biotic	AT4G39610	2.103038	0.000135	Up
	Biotic	AT1G74190	2.093231	0.00331	Up
	Biotic	AT4G28420	2.093105	4.79E-05	Up
	Biotic	AT2G47950	2.087628	0.001558	Up
	Biotic	AT3G48850	2.082971	1.33E-05	Up
	Biotic	AT4G31950	2.079134	0.000377	Up
	Biotic	AT1G17615	2.077653	0.000174	Up
	Biotic	AT4G23150	2.077439	2.73E-05	Up
	Biotic	AT5G66890	2.061989	0.000386	Up
	Biotic	AT1G26970	2.061003	0.006235	Up
	Biotic	AT1G57650	2.049015	2.57E-05	Up
	Biotic	AT2G35980	2.041576	6.67E-06	Up
	Biotic	AT3G55450	2.035884	5.15E-05	Up
	Biotic	AT1G13520	2.030538	4.14E-05	Up
	Biotic	AT4G17660	2.028918	0.004809	Up
	Biotic	AT5G13080	2.028796	9.45E-05	Up
	Biotic	AT3G13435	2.026638	0.000166	Up
	Biotic	AT3G61930	2.024049	0.004793	Up
	Biotic	AT4G04480	2.00865	0.001291	Up
	Biotic	AT1G21240	2.006397	0.002373	Up
	Biotic	AT3G44350	2.005855	0.000584	Up
	Biotic	AT5G63225	2.002857	0.000565	Up

	Biotic	AT1G51860	2.000617	0.002418	Up
	Biotic	AT2G45220	1.990432	9.12E-06	Up
	Biotic	AT3G22600	1.98709	2.32E-05	Up
	Biotic	AT5G44990	1.984321	5.78E-06	Up
	Biotic	AT3G28580	1.97711	1.29E-05	Up
	Biotic	AT5G64905	1.968588	2.09E-06	Up
	Biotic	AT2G01379	1.96813	0.008879	Up
	Biotic	AT3G47480	1.95803	2.72E-05	Up
	Biotic	AT4G22070	1.957596	0.002949	Up
	Biotic	AT1G61550	1.9538	0.000111	Up
	Biotic	AT3G13950	1.948085	0.00014	Up
	Biotic	AT1G69920	1.945559	0.003843	Up
	Biotic	AT3G61390	1.938575	0.008806	Up
	Biotic	AT1G68150	1.928997	0.001881	Up
	Biotic	AT4G18430	1.927383	4.38E-06	Up
	Biotic	AT2G19190	1.918824	2.5E-05	Up
	Biotic	AT5G53990	1.915598	0.001824	Up
	Biotic	AT1G66610	1.904713	0.005927	Up
	Biotic	AT1G75040	1.902885	0.000655	Up
	Biotic	AT1G08860	1.902077	4.52E-05	Up
	Biotic	AT4G14630	1.900535	0.000124	Up
	Biotic	AT4G11480	1.89759	8.33E-05	Up
	Biotic	AT5G15130	1.885258	0.00833	Up
	Biotic	AT5G02780	1.879536	0.000401	Up
	Biotic	AT1G68620	1.866561	8.3E-05	Up
	Biotic	AT3G46090	1.864218	0.005286	Up
	Biotic	AT1G52900	1.859726	0.004496	Up
	Biotic	AT1G51890	1.858084	1.77E-08	Up
	Biotic	AT2G39530	1.855194	9.29E-05	Up
	Biotic	AT1G33730	1.850293	0.002352	Up
	Biotic	AT4G10500	1.84891	7.54E-05	Up
	Biotic	AT4G04540	1.844797	0.008074	Up
	Biotic	AT2G14610	1.837901	0.001138	Up
	Biotic	AT2G33080	1.829261	0.000353	Up
	Biotic	AT3G07900	1.825866	0.000401	Up
	Biotic	AT2G30750	1.824366	2.92E-08	Up
	Biotic	AT5G44480	1.821275	1.81E-06	Up
	Biotic	AT3G09280	1.819345	0.004735	Up
	Biotic	AT5G48410	1.819139	0.000184	Up
	Biotic	AT2G34500	1.813608	0.000274	Up
	Biotic	AT5G64000	1.809519	0.001113	Up
	Biotic	AT1G65970	1.809094	0.000869	Up
	Biotic	AT1G36640	1.806414	7.65E-05	Up
	Biotic	AT3G57100	1.803208	0.006319	Up
	Biotic	AT1G76480	1.802927	0.005395	Up
	Biotic	AT1G53625	1.788352	0.000116	Up
	Biotic	AT5G64890	1.788181	0.00024	Up
	Biotic	AT1G65690	1.78127	1.49E-05	Up
	Biotic	AT2G40740	1.779302	0.002121	Up
	Biotic	AT2G29460	1.778419	0.000181	Up
	Biotic	AT5G42380	1.777478	0.000951	Up

	Biotic	AT5G48400	1.763284	0.000331	Up
	Biotic	AT1G26390	1.759017	1.55E-06	Up
	Biotic	AT2G18660	1.756074	0.000271	Up
	Biotic	AT4G13890	1.749671	0.004583	Up
	Biotic	AT1G70170	1.746077	0.00045	Up
	Biotic	AT1G26380	1.732755	2.45E-07	Up
	Biotic	AT5G60800	1.729624	6.67E-06	Up
	Biotic	AT4G09300	1.728776	0.0006	Up
	Biotic	AT1G34180	1.72101	0.00149	Up
	Biotic	AT5G24910	1.715333	0.00884	Up
	Biotic	AT3G01760	1.712836	0.009482	Up
	Biotic	AT1G33030	1.712623	0.000108	Up
	Biotic	AT2G46400	1.708222	0.00789	Up
	Biotic	AT5G38250	1.705703	0.006588	Up
	Biotic	AT3G21080	1.702575	2.45E-07	Up
	Biotic	AT1G72540	1.699591	5.21E-05	Up
	Biotic	AT1G69930	1.698641	0.000745	Up
	Biotic	AT2G04450	1.69785	0.007513	Up
	Biotic	AT5G26920	1.697804	7.3E-05	Up
	Biotic	AT2G33130	1.695533	0.003492	Up
	Biotic	AT4G38560	1.691773	0.000209	Up
	Biotic	AT5G67310	1.68279	0.001138	Up
	Biotic	AT1G32960	1.681008	0.000475	Up
	Biotic	AT3G46770	1.675044	0.001416	Up
	Biotic	AT1G35230	1.672779	0.001285	Up
	Biotic	AT1G21310	1.67167	0.007041	Up
	Biotic	AT2G04430	1.668787	0.000124	Up
	Biotic	AT1G47890	1.65743	0.001311	Up
	Biotic	AT1G09932	1.652259	0.000867	Up
	Biotic	AT2G23270	1.648256	0.000117	Up
	Biotic	AT4G00700	1.647854	0.009332	Up
	Biotic	AT2G43000	1.639765	1.7E-06	Up
	Biotic	AT1G13480	1.636149	0.000726	Up
	Biotic	AT1G65483	1.634492	0.000502	Up
	Biotic	AT1G07900	1.628637	3.05E-05	Up
	Biotic	AT4G23700	1.627608	1.26E-05	Up
	Biotic	AT1G74080	1.627014	7.33E-08	Up
	Biotic	AT1G02470	1.619349	0.009889	Up
	Biotic	AT5G17760	1.607242	0.00021	Up
	Biotic	AT4G14368	1.607028	0.000405	Up
	Biotic	AT4G16260	1.604504	0.000293	Up
	Biotic	AT3G57260	1.604089	0.000146	Up
	Biotic	AT5G22530	1.599649	0.000121	Up
	Biotic	AT1G14080	1.599597	0.001463	Up
	Biotic	AT1G57560	1.597681	5.84E-05	Up
	Biotic	AT4G04500	1.597077	0.004909	Up
	Biotic	AT2G34580	1.591258	0.000461	Up
	Biotic	AT3G60966	1.590164	0.000896	Up
	Biotic	AT5G54700	1.584787	0.002057	Up
	Biotic	AT3G60120	1.582596	0.00026	Up
	Biotic	AT5G51465	1.578981	0.00686	Up

	Biotic	AT4G39030	1.578548	0.001165	Up
	Biotic	AT1G09080	1.571278	0.001907	Up
	Biotic	AT3G15536	1.569125	0.000151	Up
	Biotic	AT2G24850	1.566741	0.000907	Up
	Biotic	AT3G55150	1.566467	0.000462	Up
	Biotic	AT5G52760	1.565498	0.006158	Up
	Biotic	AT3G13100	1.561185	0.000306	Up
	Biotic	AT5G01550	1.557819	0.001854	Up
	Biotic	AT4G11890	1.545624	0.000456	Up
	Biotic	AT4G14450	1.541044	0.000189	Up
	Biotic	AT1G61800	1.539778	3.33E-05	Up
	Biotic	AT1G67980	1.537729	0.000208	Up
	Biotic	AT5G22380	1.535448	0.000337	Up
	Biotic	AT3G01830	1.534053	0.001516	Up
	Biotic	AT1G66170	1.531365	0.000138	Up
	Biotic	AT2G39518	1.529204	1.26E-05	Up
	Biotic	AT4G23610	1.526483	0.001481	Up
	Biotic	AT1G14550	1.525674	0.000687	Up
	Biotic	AT3G04220	1.519593	0.004	Up
	Biotic	AT3G57460	1.518404	0.005413	Up
	Biotic	AT2G18690	1.51818	1.85E-06	Up
	Biotic	AT1G26420	1.516694	6.02E-08	Up
	Biotic	AT3G60415	1.514541	0.000204	Up
	Biotic	AT4G39670	1.51339	0.000436	Up
	Biotic	AT3G50480	1.49973	0.001439	Up
	Biotic	AT1G68765	1.498249	0.003409	Up
	Biotic	AT4G39675	1.491991	0.004134	Up
	Biotic	AT2G04495	1.483244	0.001098	Up
	Biotic	AT1G16420	1.482524	0.000253	Up
	Biotic	AT5G24110	1.480719	9.46E-05	Up
	Biotic	AT4G15417	1.470257	2.5E-05	Up
	Biotic	AT4G05020	1.466395	5.66E-06	Up
	Biotic	AT2G25440	1.463867	0.008363	Up
	Biotic	AT5G44567	1.461685	0.00218	Up
	Biotic	AT4G26120	1.460455	2.17E-08	Up
	Biotic	AT3G28210	1.453761	0.008659	Up
	Biotic	AT5G57510	1.453638	0.000482	Up
	Biotic	AT2G32460	1.451215	0.007647	Up
	Biotic	AT4G12490	1.448671	2.25E-05	Up
	Biotic	AT3G18250	1.448312	0.000138	Up
	Biotic	AT3G60961	1.44632	0.000909	Up
	Biotic	AT3G17690	1.446215	0.002756	Up
	Biotic	AT2G31865	1.442568	9.15E-05	Up
	Biotic	AT4G12500	1.442564	2.55E-06	Up
	Biotic	AT1G02920	1.438936	1.44E-07	Up
	Biotic	AT1G78410	1.426742	4.44E-05	Up
	Biotic	AT1G51920	1.426101	0.000107	Up
	Biotic	AT1G01480	1.425729	0.000462	Up
	Biotic	AT5G57010	1.422674	0.002702	Up
	Biotic	AT5G02490	1.421286	0.001024	Up
	Biotic	AT2G27389	1.416658	0.006943	Up

	Biotic	AT4G35180	1.411715	1.34E-05	Up
	Biotic	AT3G26830	1.407252	1.12E-05	Up
	Biotic	AT1G60740	1.399015	0.000252	Up
	Biotic	AT5G47850	1.398534	0.001096	Up
	Biotic	AT4G03450	1.394284	0.004321	Up
	Biotic	AT1G56060	1.379882	0.000141	Up
	Biotic	AT1G36622	1.379427	9.12E-06	Up
	Biotic	AT1G14540	1.375355	3.71E-05	Up
	Biotic	AT2G30550	1.365453	4.35E-05	Up
	Biotic	AT1G30700	1.355997	3.05E-05	Up
	Biotic	AT3G02190	1.353751	0.004791	Up
	Biotic	AT4G01720	1.351457	0.001916	Up
	Biotic	AT1G47510	1.349378	0.001523	Up
	Biotic	AT1G32940	1.348337	2.72E-05	Up
	Biotic	AT5G67340	1.345307	8.45E-05	Up
	Biotic	AT4G25070	1.342356	0.000763	Up
	Biotic	AT3G47780	1.340489	0.000329	Up
	Biotic	AT5G26170	1.333423	0.003369	Up
	Biotic	AT4G11170	1.332303	1.46E-05	Up
	Biotic	AT1G02220	1.328264	4.38E-06	Up
	Biotic	AT5G62150	1.327007	5.41E-05	Up
	Biotic	AT2G38340	1.32218	0.000327	Up
	Biotic	AT4G01540	1.319456	0.006834	Up
	Biotic	AT4G14365	1.310812	9.81E-05	Up
	Biotic	AT5G18270	1.307677	0.002606	Up
	Biotic	AT1G74590	1.303631	6.36E-05	Up
	Biotic	AT1G14870	1.300308	4.51E-06	Up
	Biotic	AT3G13090	1.297853	5.22E-05	Up
	Biotic	AT1G30900	1.297187	0.001053	Up
	Biotic	AT4G18253	1.294136	0.003979	Up
	Biotic	AT1G67520	1.293534	0.00012	Up
	Biotic	AT1G76040	1.290884	8.45E-05	Up
	Biotic	AT1G51913	1.287099	0.003235	Up
	Biotic	AT1G13470	1.285756	0.003039	Up
	Biotic	AT4G28460	1.283256	0.000542	Up
	Biotic	AT5G01250	1.28298	0.001431	Up
	Biotic	AT3G51330	1.279444	0.002131	Up
	Biotic	AT1G47980	1.279092	0.005352	Up
	Biotic	AT1G58225	1.278117	0.001043	Up
	Biotic	AT1G79450	1.27552	0.000706	Up
	Biotic	AT2G32140	1.274465	0.004497	Up
	Biotic	AT1G26240	1.27183	0.000421	Up
	Biotic	AT3G57380	1.271821	0.001896	Up
	Biotic	AT1G02930	1.270488	1.29E-05	Up
	Biotic	AT1G01560	1.269993	0.000988	Up
	Biotic	AT5G43910	1.26888	0.000688	Up
	Biotic	AT3G24900	1.268031	0.000749	Up
	Biotic	AT5G27765	1.26765	0.000311	Up
	Biotic	AT4G26470	1.267649	0.001436	Up
	Biotic	AT4G15270	1.266726	0.008867	Up
	Biotic	AT2G21910	1.262499	0.001289	Up

	Biotic	AT4G31970	1.261875	0.001358	Up
	Biotic	AT1G08050	1.258806	0.00139	Up
	Biotic	AT3G25250	1.257112	7.65E-05	Up
	Biotic	AT4G39830	1.251721	0.000736	Up
	Biotic	AT5G01900	1.251166	0.006091	Up
	Biotic	AT3G50770	1.250484	0.001979	Up
	Biotic	AT5G05340	1.2455	7.78E-05	Up
	Biotic	AT1G75170	1.244092	0.000246	Up
	Biotic	AT2G29110	1.24366	0.003023	Up
	Biotic	AT4G20110	1.240831	0.003948	Up
	Biotic	AT1G15625	1.239847	0.000465	Up
	Biotic	AT3G09940	1.236711	0.003969	Up
	Biotic	AT5G52810	1.23345	0.000395	Up
	Biotic	AT1G24145	1.233161	0.000734	Up
	Biotic	AT5G08240	1.233039	0.008848	Up
	Biotic	AT3G25510	1.230031	0.000259	Up
	Biotic	AT4G23030	1.22809	0.001613	Up
	Biotic	AT4G02520	1.224718	0.001113	Up
	Biotic	AT5G66640	1.224293	0.004092	Up
	Biotic	AT4G02380	1.223923	0.000556	Up
	Biotic	AT4G23310	1.220771	0.006536	Up
	Biotic	AT2G44370	1.217982	0.000349	Up
	Biotic	AT1G77810	1.216512	5.07E-05	Up
	Biotic	AT4G08770	1.21511	7.65E-05	Up
	Biotic	AT3G28540	1.212891	0.001516	Up
	Biotic	AT3G11640	1.211929	0.002544	Up
	Biotic	AT1G76930	1.207493	2.32E-06	Up
	Biotic	AT5G47670	1.205061	0.002078	Up
	Biotic	AT3G01970	1.204757	0.000146	Up
	Biotic	AT4G14640	1.202945	0.000147	Up
	Biotic	AT2G29750	1.202548	0.009431	Up
	Biotic	AT2G20142	1.197296	4.24E-05	Up
	Biotic	AT4G20000	1.193569	1.82E-05	Up
	Biotic	AT2G17500	1.189326	0.003655	Up
	Biotic	AT1G73810	1.188715	0.001358	Up
	Biotic	AT1G22400	1.188245	4.45E-06	Up
	Biotic	AT1G73805	1.187372	0.000441	Up
	Biotic	AT4G19370	1.187297	0.000344	Up
	Biotic	AT1G24140	1.186745	2.16E-05	Up
	Biotic	AT5G09470	1.184602	0.00541	Up
	Biotic	AT4G35380	1.184317	0.000119	Up
	Biotic	AT5G51160	1.182257	0.008572	Up
	Biotic	AT3G13380	1.181546	0.000411	Up
	Biotic	AT4G36990	1.180772	2.71E-06	Up
	Biotic	AT3G63380	1.180294	4.52E-05	Up
	Biotic	AT5G16170	1.177518	0.000874	Up
	Biotic	AT2G37980	1.175858	7.28E-05	Up
	Biotic	AT3G26500	1.174347	0.000264	Up
	Biotic	AT3G47540	1.172898	0.000465	Up
	Biotic	AT1G70690	1.16968	0.001109	Up
	Biotic	AT2G47750	1.16518	0.000181	Up

	Biotic	AT5G07650	1.163906	8.34E-06	Up
	Biotic	AT3G48020	1.162427	0.005208	Up
	Biotic	AT3G46690	1.16091	0.007394	Up
	Biotic	AT3G26470	1.16078	2.42E-05	Up
	Biotic	AT5G56960	1.160707	0.002994	Up
	Biotic	AT3G25610	1.160465	1.17E-05	Up
	Biotic	AT1G06983	1.157217	0.005706	Up
	Biotic	AT2G18680	1.15588	0.000105	Up
	Biotic	AT3G44326	1.152181	0.001545	Up
	Biotic	AT4G18250	1.151113	6.21E-05	Up
	Biotic	AT2G41380	1.147002	0.001821	Up
	Biotic	AT4G23140	1.146836	0.006178	Up
	Biotic	AT3G01420	1.146607	0.00014	Up
	Biotic	AT5G53110	1.143944	0.000199	Up
	Biotic	AT4G28390	1.143868	0.000113	Up
	Biotic	AT5G48657	1.143562	0.000125	Up
	Biotic	AT5G61490	1.139318	0.001611	Up
	Biotic	AT2G15760	1.138448	0.000252	Up
	Biotic	AT2G35730	1.131675	0.008006	Up
	Biotic	AT1G27045	1.125722	0.000368	Up
	Biotic	AT1G67810	1.124808	0.002354	Up
	Biotic	AT1G76980	1.123793	0.000141	Up
	Biotic	AT4G23210	1.121884	0.003113	Up
	Biotic	AT4G36430	1.120251	5.48E-07	Up
	Biotic	AT4G26270	1.11965	9.81E-05	Up
	Biotic	AT4G22670	1.117474	0.009286	Up
	Biotic	AT2G04070	1.116127	0.000421	Up
	Biotic	AT3G44830	1.104863	0.000858	Up
	Biotic	AT2G25297	1.104742	0.00012	Up
	Biotic	AT1G64065	1.104021	0.000569	Up
	Biotic	AT2G44290	1.103747	1.81E-06	Up
	Biotic	AT1G07620	1.102969	0.000167	Up
	Biotic	AT2G38860	1.098112	7.57E-06	Up
	Biotic	AT3G05500	1.097676	2.63E-05	Up
	Biotic	AT4G37390	1.095828	0.007521	Up
	Biotic	AT2G28710	1.095475	8.28E-05	Up
	Biotic	AT4G23280	1.092032	0.000103	Up
	Biotic	AT2G17740	1.089929	2.72E-05	Up
	Biotic	AT1G66410	1.087874	0.000421	Up
	Biotic	AT2G45570	1.08762	0.003891	Up
	Biotic	AT3G26840	1.087361	1.08E-05	Up
	Biotic	AT5G45000	1.084896	0.00466	Up
	Biotic	AT3G22160	1.084207	1.22E-05	Up
	Biotic	AT5G24210	1.082467	1.26E-05	Up
	Biotic	AT1G60730	1.082249	0.000105	Up
	Biotic	AT1G07160	1.081487	0.001626	Up
	Biotic	AT1G02520	1.080656	0.000225	Up
	Biotic	AT4G26990	1.080546	0.000129	Up
	Biotic	AT4G07820	1.075641	0.000012	Up
	Biotic	AT3G52340	1.075321	8.87E-07	Up
	Biotic	AT5G58940	1.074611	9.12E-06	Up

	Biotic	AT3G54150	1.073883	0.000842	Up
	Biotic	AT5G28646	1.067422	0.006842	Up
	Biotic	AT1G69520	1.067404	0.000377	Up
	Biotic	AT2G37620	1.065064	0.002292	Up
	Biotic	AT4G17670	1.064348	0.001966	Up
	Biotic	AT5G42010	1.061331	0.000788	Up
	Biotic	AT5G20960	1.059203	0.00263	Up
	Biotic	AT1G48605	1.054523	9.31E-05	Up
	Biotic	AT4G37900	1.053415	0.000104	Up
	Biotic	AT3G26210	1.050086	0.003866	Up
	Biotic	AT1G67920	1.047635	0.003967	Up
	Biotic	AT5G07780	1.046389	0.004918	Up
	Biotic	AT2G38250	1.045102	0.0008	Up
	Biotic	AT3G11000	1.043443	0.000201	Up
	Biotic	AT1G64610	1.042753	2.5E-05	Up
	Biotic	AT5G59530	1.037645	0.00013	Up
	Biotic	AT1G63840	1.037526	0.000182	Up
	Biotic	AT4G10960	1.036391	0.00011	Up
	Biotic	AT5G64120	1.026806	1.07E-05	Up
	Biotic	AT1G51420	1.026407	0.000245	Up
	Biotic	AT3G09010	1.025679	0.000684	Up
	Biotic	AT5G24090	1.023572	0.000225	Up
	Biotic	AT3G12910	1.021173	0.001107	Up
	Biotic	AT2G47000	1.020643	7.89E-05	Up
	Biotic	AT5G46230	1.018154	0.006221	Up
	Biotic	AT2G29990	1.016202	0.000225	Up
	Biotic	AT2G43620	1.015706	1.81E-06	Up
	Biotic	AT1G10417	1.012983	0.006668	Up
	Biotic	AT3G23600	1.00708	0.002912	Up
	Biotic	AT3G08970	1.006956	0.002878	Up
	Biotic	AT5G25820	1.006618	0.001394	Up
	Biotic	AT4G37030	1.003489	0.00054	Up
	Biotic	AT3G52460	1.002525	0.000132	Up
	Biotic	AT5G57480	1.001412	0.000141	Up
	Biotic	AT1G12290	1.000513	0.000122	Up
	Biotic	AT2G29300	-1.01664	0.002405	Down
	Biotic	AT5G65730	-1.02736	0.00054	Down
	Biotic	AT1G59720	-1.04258	0.008107	Down
	Biotic	AT5G10250	-1.07941	2.13E-05	Down
	Biotic	AT5G25240	-1.11364	0.005128	Down
	Biotic	AT2G23690	-1.20432	0.000972	Down
	Biotic	AT1G50040	-1.28795	0.007874	Down
	Biotic	AT1G13650	-1.29366	0.000129	Down
	Biotic	AT5G24640	-1.33816	0.005651	Down
	Biotic	AT2G20670	-1.33837	0.000163	Down
	Biotic	AT1G62510	-1.342	1.35E-05	Down
	Biotic	AT4G08950	-1.3784	3.8E-05	Down
	Biotic	AT2G27420	-1.41201	0.000157	Down
	Biotic	AT5G62730	-1.43547	0.009907	Down
	Biotic	AT3G48360	-1.49299	0.001403	Down
	Biotic	AT5G08150	-1.55175	0.000556	Down

	Biotic	AT1G21910	-1.62105	1.15E-05	Down
	Biotic	AT3G45970	-1.71122	4.32E-08	Down
	Biotic	AT5G57560	-2.11846	0.000159	Down
	Biotic	AT1G35140	-2.80687	0.000617	Down
	Biotic	AT2G33847	-3.78792	0.004733	Down
	Abiotic	AT1G01470	1.412094	2.28E-06	Up
	Abiotic	AT1G01720	1.360304	1.94E-06	Up
	Abiotic	AT1G01810	1.470931	2.72E-07	Up
	Abiotic	AT1G01940	1.053412	4.04E-06	Up
	Abiotic	AT1G02300	1.05793	4.23E-08	Up
	Abiotic	AT1G02390	1.360029	7.84E-08	Up
	Abiotic	AT1G02980	1.323106	4.22E-05	Up
	Abiotic	AT1G04570	1.979582	2.13E-14	Up
	Abiotic	AT1G05340	1.166264	0.000176	Up
	Abiotic	AT1G07330	1.546117	1.87E-06	Up
	Abiotic	AT1G07400	1.413668	2.84E-06	Up
	Abiotic	AT1G07430	2.291214	2.14E-11	Up
	Abiotic	AT1G07900	1.081077	0.000505	Up
	Abiotic	AT1G08440	1.462686	1.02E-05	Up
	Abiotic	AT1G09157	1.118113	0.000262	Up
	Abiotic	AT1G09950	2.372008	1.67E-12	Up
	Abiotic	AT1G10170	1.268525	1.28E-08	Up
	Abiotic	AT1G10560	1.080764	7.99E-05	Up
	Abiotic	AT1G11210	1.209469	0.000122	Up
	Abiotic	AT1G12290	1.256694	1.93E-07	Up
	Abiotic	AT1G13370	1.011361	9.99E-05	Up
	Abiotic	AT1G14205	1.11352	4.41E-06	Up
	Abiotic	AT1G14360	1.154751	4.49E-07	Up
	Abiotic	AT1G14880	1.205301	0.000168	Up
	Abiotic	AT1G15580	1.028729	0.000121	Up
	Abiotic	AT1G16850	1.883867	2.42E-08	Up
	Abiotic	AT1G17380	1.437237	9.96E-07	Up
	Abiotic	AT1G17420	1.068209	1.86E-05	Up
	Abiotic	AT1G18830	1.26401	2.09E-05	Up
	Abiotic	AT1G19180	1.153287	0.000104	Up
	Abiotic	AT1G20150	1.102255	0.000404	Up
	Abiotic	AT1G20440	1.264115	8.54E-06	Up
	Abiotic	AT1G20450	1.539082	3.88E-08	Up
	Abiotic	AT1G22110	1.335377	1.46E-05	Up
	Abiotic	AT1G22370	1.041949	2.23E-06	Up
	Abiotic	AT1G27730	1.036076	0.0008	Up
	Abiotic	AT1G28370	1.061659	0.000469	Up
	Abiotic	AT1G29395	1.199723	7.77E-05	Up
	Abiotic	AT1G29640	1.076143	0.000171	Up
	Abiotic	AT1G30135	1.428899	1.03E-05	Up
	Abiotic	AT1G30190	1.509482	2.57E-06	Up
	Abiotic	AT1G31243	1.102553	0.00046	Up
	Abiotic	AT1G32870	1.231998	4.51E-08	Up
	Abiotic	AT1G32900	1.120188	0.000103	Up
	Abiotic	AT1G35720	1.141944	1.62E-05	Up
	Abiotic	AT1G35730	2.324076	1.71E-11	Up

	Abiotic	AT1G45145	1.082066	0.000298	Up
	Abiotic	AT1G47510	1.217084	0.000109	Up
	Abiotic	AT1G48720	1.78324	1.79E-07	Up
	Abiotic	AT1G50400	1.082031	2.06E-06	Up
	Abiotic	AT1G50780	1.188516	0.000211	Up
	Abiotic	AT1G52560	1.230344	7.37E-05	Up
	Abiotic	AT1G52690	1.049219	0.000535	Up
	Abiotic	AT1G52890	1.507434	6.29E-06	Up
	Abiotic	AT1G53540	1.702725	3.70E-07	Up
	Abiotic	AT1G53885	1.244554	8.50E-05	Up
	Abiotic	AT1G53903	1.032248	0.000842	Up
	Abiotic	AT1G54040	1.141391	1.91E-05	Up
	Abiotic	AT1G54160	1.267694	4.63E-10	Up
	Abiotic	AT1G54773	1.036977	1.47E-05	Up
	Abiotic	AT1G56300	1.310266	9.42E-06	Up
	Abiotic	AT1G56600	1.227115	9.28E-05	Up
	Abiotic	AT1G56650	1.124154	5.07E-05	Up
	Abiotic	AT1G59865	1.8717	3.02E-09	Up
	Abiotic	AT1G60190	1.992208	2.86E-09	Up
	Abiotic	AT1G61340	1.849008	4.60E-10	Up
	Abiotic	AT1G61470	1.118692	8.70E-07	Up
	Abiotic	AT1G61800	2.496775	2.56E-13	Up
	Abiotic	AT1G62290	1.213242	2.48E-05	Up
	Abiotic	AT1G62710	1.225198	1.56E-05	Up
	Abiotic	AT1G63550	1.046426	0.000445	Up
	Abiotic	AT1G63570	1.022219	0.000902	Up
	Abiotic	AT1G63730	1.102463	9.71E-09	Up
	Abiotic	AT1G64200	1.108849	4.77E-06	Up
	Abiotic	AT1G64210	1.006854	0.000612	Up
	Abiotic	AT1G64360	1.131972	0.000339	Up
	Abiotic	AT1G64561	1.029882	0.000112	Up
	Abiotic	AT1G64563	2.122364	5.14E-12	Up
	Abiotic	AT1G65890	1.116714	0.00024	Up
	Abiotic	AT1G66500	2.325331	1.30E-15	Up
	Abiotic	AT1G66510	1.173192	2.24E-07	Up
	Abiotic	AT1G67220	1.278817	7.25E-05	Up
	Abiotic	AT1G67360	2.57439	6.18E-17	Up
	Abiotic	AT1G67370	1.17609	9.83E-06	Up
	Abiotic	AT1G67623	1.322649	4.58E-05	Up
	Abiotic	AT1G67920	1.64455	4.83E-07	Up
	Abiotic	AT1G68050	1.009026	0.00085	Up
	Abiotic	AT1G68300	1.273905	3.27E-07	Up
	Abiotic	AT1G68765	1.02143	0.000896	Up
	Abiotic	AT1G68930	1.073771	4.84E-08	Up
	Abiotic	AT1G69490	1.264256	3.16E-05	Up
	Abiotic	AT1G69790	1.012376	0.000914	Up
	Abiotic	AT1G70300	1.00072	2.92E-05	Up
	Abiotic	AT1G70640	1.948032	2.79E-09	Up
	Abiotic	AT1G70800	1.567129	1.08E-07	Up
	Abiotic	AT1G71000	1.458847	1.13E-05	Up
	Abiotic	AT1G72660	3.26531	3.36E-23	Up

	Abiotic	AT1G72760	1.656339	2.13E-08	Up
	Abiotic	AT1G72900	1.149418	2.09E-05	Up
	Abiotic	AT1G73066	1.168472	4.52E-07	Up
	Abiotic	AT1G73480	1.438863	5.99E-07	Up
	Abiotic	AT1G73880	1.066387	6.98E-07	Up
	Abiotic	AT1G74930	1.94941	8.79E-12	Up
	Abiotic	AT1G75600	1.555927	3.04E-06	Up
	Abiotic	AT1G76590	1.728603	6.48E-09	Up
	Abiotic	AT1G76650	1.086642	5.95E-05	Up
	Abiotic	AT1G77000	1.587578	1.16E-09	Up
	Abiotic	AT1G77120	1.539463	1.84E-06	Up
	Abiotic	AT1G78070	1.470111	1.23E-08	Up
	Abiotic	AT1G78380	1.191313	7.35E-07	Up
	Abiotic	AT1G78390	1.448023	1.19E-05	Up
	Abiotic	AT1G78680	1.132498	8.79E-07	Up
	Abiotic	AT1G79920	1.107758	2.89E-07	Up
	Abiotic	AT1G80110	1.113079	6.13E-07	Up
	Abiotic	AT1G80120	1.150504	3.37E-05	Up
	Abiotic	AT1G80130	1.584303	1.04E-06	Up
	Abiotic	AT1G80660	1.525226	3.91E-07	Up
	Abiotic	AT2G01008	2.353944	4.93E-12	Up
	Abiotic	AT2G07776	1.289219	2.16E-05	Up
	Abiotic	AT2G07749	1.006202	0.00111	Up
	Abiotic	AT2G07777	1.666294	6.47E-08	Up
	Abiotic	AT2G07671	1.330315	2.49E-05	Up
	Abiotic	AT2G07779	1.634476	9.87E-07	Up
	Abiotic	AT2G07672	1.637997	1.26E-06	Up
	Abiotic	AT2G07674	1.238647	0.000104	Up
	Abiotic	AT2G07678	1.834545	5.35E-08	Up
	Abiotic	AT2G07774	1.36831	3.09E-06	Up
	Abiotic	AT2G07687	1.341501	2.98E-05	Up
	Abiotic	AT2G07692	1.460353	1.15E-05	Up
	Abiotic	AT2G07695	1.362013	2.12E-05	Up
	Abiotic	AT2G07785	1.436726	7.29E-06	Up
	Abiotic	AT2G07599	1.006188	0.001101	Up
	Abiotic	AT2G07798	1.216732	0.000155	Up
	Abiotic	AT2G07698	1.822038	2.55E-08	Up
	Abiotic	AT2G07702	1.097551	0.000202	Up
	Abiotic	AT2G07706	1.109644	0.000438	Up
	Abiotic	AT2G07715	1.037431	7.25E-05	Up
	Abiotic	AT2G07648	1.087797	0.000361	Up
	Abiotic	AT2G07719	1.3497	1.85E-05	Up
	Abiotic	AT2G07652	1.441166	1.35E-05	Up
	Abiotic	AT2G07815	1.549459	8.57E-07	Up
	Abiotic	AT2G07724	1.422949	1.74E-05	Up
	Abiotic	AT2G07725	1.531272	2.22E-06	Up
	Abiotic	AT2G07728	1.139667	0.000254	Up
	Abiotic	AT2G07734	1.191911	0.000205	Up
	Abiotic	AT2G07775	1.271552	8.95E-05	Up
	Abiotic	AT2G07661	1.153292	0.000276	Up
	Abiotic	AT2G05365	1.131405	0.000229	Up

	Abiotic	AT2G07738	1.138682	0.000168	Up
	Abiotic	AT2G07795	1.190511	0.000163	Up
	Abiotic	AT2G07739	1.023946	0.000803	Up
	Abiotic	AT2G07662	1.090324	0.000507	Up
	Abiotic	AT2G07835	1.245422	0.00012	Up
	Abiotic	AT2G13960	1.234176	3.36E-07	Up
	Abiotic	AT2G15790	1.058001	2.37E-06	Up
	Abiotic	AT2G16586	1.356491	1.89E-06	Up
	Abiotic	AT2G17660	1.44568	3.12E-06	Up
	Abiotic	AT2G18680	1.208105	7.90E-07	Up
	Abiotic	AT2G19900	1.040561	0.000752	Up
	Abiotic	AT2G20560	1.039381	9.59E-05	Up
	Abiotic	AT2G20825	1.046532	0.000788	Up
	Abiotic	AT2G20880	1.454053	6.55E-06	Up
	Abiotic	AT2G21130	1.58525	1.51E-09	Up
	Abiotic	AT2G21640	1.325919	1.64E-08	Up
	Abiotic	AT2G21660	1.215408	1.53E-05	Up
	Abiotic	AT2G21780	1.304874	1.03E-05	Up
	Abiotic	AT2G21940	1.010405	1.54E-07	Up
	Abiotic	AT2G22470	1.367472	1.51E-05	Up
	Abiotic	AT2G24940	1.136954	2.86E-06	Up
	Abiotic	AT2G25140	1.20775	2.79E-07	Up
	Abiotic	AT2G25460	1.62	1.06E-08	Up
	Abiotic	AT2G26150	1.211499	1.91E-05	Up
	Abiotic	AT2G27280	1.003248	0.001086	Up
	Abiotic	AT2G28400	1.435944	7.82E-08	Up
	Abiotic	AT2G28900	1.526893	4.33E-08	Up
	Abiotic	AT2G29450	1.421997	1.04E-08	Up
	Abiotic	AT2G29500	2.866581	1.44E-16	Up
	Abiotic	AT2G30830	1.191221	2.76E-05	Up
	Abiotic	AT2G31590	1.34123	1.62E-05	Up
	Abiotic	AT2G31910	1.076823	8.16E-05	Up
	Abiotic	AT2G32120	1.168086	1.42E-06	Up
	Abiotic	AT2G32140	1.282923	7.13E-05	Up
	Abiotic	AT2G32190	1.18132	7.74E-05	Up
	Abiotic	AT2G32210	1.236878	1.52E-05	Up
	Abiotic	AT2G33380	1.83829	2.25E-09	Up
	Abiotic	AT2G33580	1.117651	3.81E-07	Up
	Abiotic	AT2G33590	1.726104	1.84E-10	Up
	Abiotic	AT2G34810	1.262748	6.51E-06	Up
	Abiotic	AT2G36220	1.631661	4.41E-07	Up
	Abiotic	AT2G36460	1.507786	2.93E-09	Up
	Abiotic	AT2G36780	1.123355	0.000377	Up
	Abiotic	AT2G37180	1.33863	2.44E-08	Up
	Abiotic	AT2G37870	1.142629	0.000328	Up
	Abiotic	AT2G38255	1.256957	7.38E-05	Up
	Abiotic	AT2G38340	2.076644	2.02E-11	Up
	Abiotic	AT2G38465	1.017406	0.000444	Up
	Abiotic	AT2G39050	1.248362	3.54E-07	Up
	Abiotic	AT2G39420	1.036886	9.81E-05	Up
	Abiotic	AT2G39800	1.006321	2.40E-05	Up

	Abiotic	AT2G40340	1.976764	3.16E-09	Up
	Abiotic	AT2G41190	1.213712	1.88E-06	Up
	Abiotic	AT2G42530	1.938671	3.37E-09	Up
	Abiotic	AT2G42540	2.36602	8.31E-12	Up
	Abiotic	AT2G43020	1.03706	1.48E-06	Up
	Abiotic	AT2G44578	1.276639	7.40E-05	Up
	Abiotic	AT2G45350	1.455711	1.81E-07	Up
	Abiotic	AT2G45760	1.243727	0.000122	Up
	Abiotic	AT2G45840	1.064179	0.000482	Up
	Abiotic	AT2G45940	1.156443	0.000164	Up
	Abiotic	AT2G47180	1.561526	6.94E-07	Up
	Abiotic	AT2G47520	1.15966	0.000116	Up
	Abiotic	AT2G47780	1.214353	2.96E-07	Up
	Abiotic	AT3G03260	1.35195	3.45E-05	Up
	Abiotic	AT3G03270	1.028025	0.000186	Up
	Abiotic	AT3G03341	1.763994	2.27E-07	Up
	Abiotic	AT3G04050	1.0978	4.98E-05	Up
	Abiotic	AT3G04710	1.436176	1.70E-08	Up
	Abiotic	AT3G05640	1.454533	1.23E-08	Up
	Abiotic	AT3G05650	1.298449	6.21E-06	Up
	Abiotic	AT3G05880	1.068198	4.07E-05	Up
	Abiotic	AT3G08690	1.218521	1.28E-07	Up
	Abiotic	AT3G08700	1.368582	3.27E-05	Up
	Abiotic	AT3G08970	1.036071	2.33E-05	Up
	Abiotic	AT3G09390	1.156031	2.63E-05	Up
	Abiotic	AT3G09640	1.256009	6.24E-05	Up
	Abiotic	AT3G10560	1.915734	1.18E-08	Up
	Abiotic	AT3G11020	2.180431	1.97E-11	Up
	Abiotic	AT3G11410	1.18004	1.56E-08	Up
	Abiotic	AT3G11580	1.325064	5.56E-07	Up
	Abiotic	AT3G12580	1.265289	5.32E-05	Up
	Abiotic	AT3G13130	1.179491	0.000221	Up
	Abiotic	AT3G14200	1.42266	1.05E-08	Up
	Abiotic	AT3G14440	1.502983	1.55E-06	Up
	Abiotic	AT3G15740	1.015683	0.000213	Up
	Abiotic	AT3G18360	1.043332	0.00018	Up
	Abiotic	AT3G18810	1.137764	0.000222	Up
	Abiotic	AT3G19270	1.062892	4.07E-05	Up
	Abiotic	AT3G19580	1.115462	5.56E-05	Up
	Abiotic	AT3G19920	1.39231	2.48E-05	Up
	Abiotic	AT3G21660	1.147564	0.000103	Up
	Abiotic	AT3G21890	1.355928	1.45E-05	Up
	Abiotic	AT3G22090	1.627667	4.66E-07	Up
	Abiotic	AT3G22100	1.947906	2.36E-09	Up
	Abiotic	AT3G22275	1.218637	0.000153	Up
	Abiotic	AT3G22840	1.256384	4.40E-05	Up
	Abiotic	AT3G25230	1.322285	1.80E-08	Up
	Abiotic	AT3G25240	1.098677	0.000408	Up
	Abiotic	AT3G28210	1.160312	0.000125	Up
	Abiotic	AT3G29575	1.006736	5.33E-05	Up
	Abiotic	AT3G41762	1.520019	3.01E-06	Up

	Abiotic	AT3G44260	1.633625	8.79E-08	Up
	Abiotic	AT3G45940	1.677682	3.14E-07	Up
	Abiotic	AT3G46640	1.076603	1.21E-05	Up
	Abiotic	AT3G48240	1.232348	1.29E-05	Up
	Abiotic	AT3G48520	1.197029	0.000145	Up
	Abiotic	AT3G48835	1.322857	4.66E-06	Up
	Abiotic	AT3G50940	1.085608	0.000395	Up
	Abiotic	AT3G53230	1.906835	2.25E-10	Up
	Abiotic	AT3G53990	1.517361	7.54E-08	Up
	Abiotic	AT3G54950	1.103095	2.47E-06	Up
	Abiotic	AT3G55970	1.221835	0.000142	Up
	Abiotic	AT3G55980	1.10044	2.34E-06	Up
	Abiotic	AT3G56250	1.079927	9.09E-06	Up
	Abiotic	AT3G56790	1.619626	1.01E-06	Up
	Abiotic	AT3G59730	1.181714	7.32E-05	Up
	Abiotic	AT3G62260	1.585979	1.14E-09	Up
	Abiotic	AT3G63060	1.244757	4.23E-08	Up
	Abiotic	AT4G00940	1.274839	1.24E-06	Up
	Abiotic	AT4G01360	1.131028	0.000214	Up
	Abiotic	AT4G01435	1.379709	1.81E-05	Up
	Abiotic	AT4G01550	1.129503	2.14E-07	Up
	Abiotic	AT4G02005	1.371486	1.04E-08	Up
	Abiotic	AT4G02650	1.043338	0.000811	Up
	Abiotic	AT4G02690	1.163975	0.000269	Up
	Abiotic	AT4G03320	1.355805	1.01E-06	Up
	Abiotic	AT4G03430	1.274488	3.24E-10	Up
	Abiotic	AT4G03930	1.124812	5.85E-05	Up
	Abiotic	AT4G04020	2.03889	2.62E-11	Up
	Abiotic	AT4G09020	1.326822	2.85E-07	Up
	Abiotic	AT4G09130	1.178561	0.000212	Up
	Abiotic	AT4G10843	1.107389	5.96E-06	Up
	Abiotic	AT4G11660	1.131455	2.38E-07	Up
	Abiotic	AT4G12290	1.157924	2.21E-05	Up
	Abiotic	AT4G12400	1.241998	7.60E-06	Up
	Abiotic	AT4G13395	1.461733	7.52E-06	Up
	Abiotic	AT4G13800	1.139701	2.79E-05	Up
	Abiotic	AT4G15120	1.044451	0.000123	Up
	Abiotic	AT4G16500	1.067233	1.37E-06	Up
	Abiotic	AT4G17230	1.044486	1.92E-06	Up
	Abiotic	AT4G17470	1.501299	1.03E-06	Up
	Abiotic	AT4G18280	1.697466	4.54E-09	Up
	Abiotic	AT4G18980	1.187545	0.000173	Up
	Abiotic	AT4G19230	1.376727	4.65E-07	Up
	Abiotic	AT4G19430	1.962186	5.90E-09	Up
	Abiotic	AT4G21320	1.145832	8.46E-06	Up
	Abiotic	AT4G21440	1.259779	5.10E-05	Up
	Abiotic	AT4G21680	1.364937	2.41E-05	Up
	Abiotic	AT4G23600	1.134361	0.000132	Up
	Abiotic	AT4G24380	1.034066	2.30E-06	Up
	Abiotic	AT4G24410	1.049819	0.00021	Up
	Abiotic	AT4G24413	1.985235	8.98E-09	Up

	Abiotic	AT4G24570	1.928306	7.15E-12	Up
	Abiotic	AT4G25000	1.386716	2.05E-05	Up
	Abiotic	AT4G25340	1.074699	1.35E-05	Up
	Abiotic	AT4G25380	1.206477	0.000147	Up
	Abiotic	AT4G25433	1.217341	0.000118	Up
	Abiotic	AT4G25480	1.395791	9.24E-06	Up
	Abiotic	AT4G25490	1.107888	0.000439	Up
	Abiotic	AT4G25810	1.020668	0.000155	Up
	Abiotic	AT4G26080	1.127214	8.11E-08	Up
	Abiotic	AT4G26270	1.908057	5.96E-11	Up
	Abiotic	AT4G26780	1.463703	5.47E-08	Up
	Abiotic	AT4G27410	1.567787	1.83E-07	Up
	Abiotic	AT4G27654	1.36337	3.07E-05	Up
	Abiotic	AT4G28140	1.723826	1.79E-07	Up
	Abiotic	AT4G28390	1.437726	8.65E-07	Up
	Abiotic	AT4G29770	2.031179	1.24E-09	Up
	Abiotic	AT4G29780	1.352816	1.58E-07	Up
	Abiotic	AT4G31351	1.289076	2.88E-05	Up
	Abiotic	AT4G31354	1.769308	1.24E-08	Up
	Abiotic	AT4G31405	1.537782	2.07E-06	Up
	Abiotic	AT4G31408	1.207748	1.02E-06	Up
	Abiotic	AT4G32450	1.085198	1.60E-06	Up
	Abiotic	AT4G33467	1.136471	0.0003	Up
	Abiotic	AT4G33905	1.972294	2.46E-10	Up
	Abiotic	AT4G33930	1.74381	2.58E-07	Up
	Abiotic	AT4G33940	1.095128	8.92E-07	Up
	Abiotic	AT4G33985	1.489935	1.81E-08	Up
	Abiotic	AT4G34850	1.075841	7.67E-05	Up
	Abiotic	AT4G35690	1.113521	0.000431	Up
	Abiotic	AT4G35985	1.55564	5.02E-08	Up
	Abiotic	AT4G36010	1.792834	2.37E-09	Up
	Abiotic	AT4G36490	1.530089	5.00E-06	Up
	Abiotic	AT4G36795	1.062137	0.000618	Up
	Abiotic	AT4G36950	1.262703	9.84E-05	Up
	Abiotic	AT4G36990	1.972688	4.52E-10	Up
	Abiotic	AT4G39110	1.747989	5.54E-10	Up
	Abiotic	AT4G39210	1.414439	3.91E-08	Up
	Abiotic	AT4G39360	1.21101	0.000133	Up
	Abiotic	AT5G01520	1.388574	5.18E-07	Up
	Abiotic	AT5G01990	1.528958	1.36E-11	Up
	Abiotic	AT5G02020	1.181495	7.28E-05	Up
	Abiotic	AT5G03020	1.098189	0.000162	Up
	Abiotic	AT5G03204	1.435903	8.54E-06	Up
	Abiotic	AT5G03210	1.816079	7.72E-08	Up
	Abiotic	AT5G03435	1.078795	0.000352	Up
	Abiotic	AT5G03720	1.50087	2.12E-08	Up
	Abiotic	AT5G04250	1.1913	2.06E-09	Up
	Abiotic	AT5G04340	1.706695	2.69E-07	Up
	Abiotic	AT5G05220	2.015258	4.76E-09	Up
	Abiotic	AT5G05410	3.103058	5.40E-19	Up
	Abiotic	AT5G05490	1.17667	1.67E-05	Up

	Abiotic	AT5G10340	1.6157	1.00E-06	Up
	Abiotic	AT5G11680	1.520538	3.64E-09	Up
	Abiotic	AT5G12020	1.005464	0.000921	Up
	Abiotic	AT5G12110	1.399979	1.75E-07	Up
	Abiotic	AT5G13200	1.330518	7.67E-06	Up
	Abiotic	AT5G13220	1.015283	1.71E-06	Up
	Abiotic	AT5G15450	1.57187	8.67E-10	Up
	Abiotic	AT5G15537	1.121426	1.94E-06	Up
	Abiotic	AT5G15960	1.514799	5.91E-06	Up
	Abiotic	AT5G15970	1.778547	8.49E-09	Up
	Abiotic	AT5G16380	1.09786	4.44E-07	Up
	Abiotic	AT5G17460	1.737152	1.02E-09	Up
	Abiotic	AT5G17760	1.130294	5.97E-06	Up
	Abiotic	AT5G17850	1.016405	6.04E-07	Up
	Abiotic	AT5G19720	1.053157	0.000742	Up
	Abiotic	AT5G19875	1.084078	4.49E-05	Up
	Abiotic	AT5G22290	1.136559	4.10E-07	Up
	Abiotic	AT5G24600	1.12379	0.00034	Up
	Abiotic	AT5G25110	1.15151	6.83E-05	Up
	Abiotic	AT5G25280	1.847772	8.21E-10	Up
	Abiotic	AT5G25390	1.320386	2.46E-05	Up
	Abiotic	AT5G25450	1.494968	2.73E-06	Up
	Abiotic	AT5G25755	1.643518	6.08E-09	Up
	Abiotic	AT5G25756	1.304071	2.34E-05	Up
	Abiotic	AT5G04065	1.532921	4.07E-06	Up
	Abiotic	AT5G27280	1.032764	3.73E-06	Up
	Abiotic	AT5G28237	1.526409	5.24E-06	Up
	Abiotic	AT5G37340	1.116935	5.80E-08	Up
	Abiotic	AT5G37440	1.384356	3.16E-08	Up
	Abiotic	AT5G37750	1.339777	3.29E-06	Up
	Abiotic	AT5G05545	1.083192	6.69E-05	Up
	Abiotic	AT5G37940	1.146765	0.000289	Up
	Abiotic	AT5G38130	1.341605	1.73E-05	Up
	Abiotic	AT5G38895	1.114225	1.67E-05	Up
	Abiotic	AT5G40382	1.221298	0.000145	Up
	Abiotic	AT5G40790	1.074057	0.000546	Up
	Abiotic	AT5G42380	1.027083	0.000875	Up
	Abiotic	AT5G42900	1.107347	0.000426	Up
	Abiotic	AT5G43300	1.117619	0.000218	Up
	Abiotic	AT5G43440	1.178819	4.42E-05	Up
	Abiotic	AT5G43620	2.073894	2.62E-13	Up
	Abiotic	AT5G43950	1.213208	1.15E-09	Up
	Abiotic	AT5G44005	1.622541	4.27E-09	Up
	Abiotic	AT5G45630	1.959202	1.35E-08	Up
	Abiotic	AT5G46040	1.110766	0.000427	Up
	Abiotic	AT5G48570	2.277575	2.01E-13	Up
	Abiotic	AT5G49200	1.376008	3.01E-05	Up
	Abiotic	AT5G49920	1.071702	0.000297	Up
	Abiotic	AT5G50800	1.098711	0.000391	Up
	Abiotic	AT5G51440	1.514646	2.37E-07	Up
	Abiotic	AT5G51990	1.056197	0.000647	Up

	Abiotic	AT5G52300	1.052717	0.000746	Up
	Abiotic	AT5G52310	2.009743	2.56E-10	Up
	Abiotic	AT5G52630	1.273237	2.45E-08	Up
	Abiotic	AT5G52640	1.889731	2.59E-09	Up
	Abiotic	AT5G53710	1.008682	0.000996	Up
	Abiotic	AT5G53740	1.18583	0.000218	Up
	Abiotic	AT5G53870	1.000996	0.000107	Up
	Abiotic	AT5G54165	1.240637	0.000117	Up
	Abiotic	AT5G54940	1.213682	4.93E-06	Up
	Abiotic	AT5G55050	1.066436	0.000297	Up
	Abiotic	AT5G56010	1.07004	1.35E-05	Up
	Abiotic	AT5G56600	1.148713	9.49E-08	Up
	Abiotic	AT5G57050	1.221872	1.50E-09	Up
	Abiotic	AT5G57150	1.007172	2.45E-07	Up
	Abiotic	AT5G57510	1.186421	0.000216	Up
	Abiotic	AT5G57560	1.375362	1.93E-07	Up
	Abiotic	AT5G58787	1.108309	2.01E-06	Up
	Abiotic	AT5G59220	1.279098	1.96E-05	Up
	Abiotic	AT5G59310	1.437859	1.37E-05	Up
	Abiotic	AT5G59320	1.31432	5.73E-05	Up
	Abiotic	AT5G59720	3.289387	1.64E-20	Up
	Abiotic	AT5G59820	1.959792	1.02E-08	Up
	Abiotic	AT5G60320	1.150295	0.000248	Up
	Abiotic	AT5G61890	1.07724	0.000225	Up
	Abiotic	AT5G62040	1.277856	6.32E-05	Up
	Abiotic	AT5G62520	2.843117	1.50E-16	Up
	Abiotic	AT5G63130	2.098882	6.93E-13	Up
	Abiotic	AT5G64230	1.232633	2.56E-06	Up
	Abiotic	AT5G64510	2.71291	2.03E-15	Up
	Abiotic	AT5G64750	1.047183	0.000364	Up
	Abiotic	AT5G64870	1.447589	2.29E-08	Up
	Abiotic	AT5G65300	1.593605	1.92E-08	Up
	Abiotic	AT5G66110	1.178404	8.14E-05	Up
	Abiotic	AT5G66400	1.137459	0.000345	Up
	Abiotic	AT5G66880	1.173685	2.08E-07	Up
	Abiotic	AT5G67290	1.076177	9.82E-07	Up
	Abiotic	AT5G67310	1.409404	1.62E-05	Up
	Abiotic	AT5G67480	1.434107	4.50E-07	Up
	Abiotic	ATMG00040	1.233791	0.00013	Up
	Abiotic	ATMG00060	2.438736	7.89E-13	Up
	Abiotic	ATMG00070	1.461416	3.42E-06	Up
	Abiotic	ATMG00080	1.345877	2.39E-05	Up
	Abiotic	ATMG00090	1.122366	0.000262	Up
	Abiotic	ATMG00110	1.54814	9.74E-07	Up
	Abiotic	ATMG00160	2.138813	8.64E-11	Up
	Abiotic	ATMG00270	2.108584	1.06E-09	Up
	Abiotic	ATMG00285	1.908618	1.60E-08	Up
	Abiotic	ATMG00300	1.355056	3.30E-05	Up
	Abiotic	ATMG00400	1.034836	0.000805	Up
	Abiotic	ATMG00410	1.13493	0.000339	Up
	Abiotic	ATMG00450	1.189067	0.000142	Up

	Abiotic	ATMG00510	2.048819	1.50E-10	Up
	Abiotic	ATMG00513	1.853702	1.89E-08	Up
	Abiotic	ATMG00516	1.646964	7.81E-07	Up
	Abiotic	ATMG00560	1.600818	1.76E-06	Up
	Abiotic	ATMG00630	1.231342	8.13E-05	Up
	Abiotic	ATMG00640	1.726131	7.90E-08	Up
	Abiotic	ATMG00650	2.061594	2.61E-09	Up
	Abiotic	ATMG00660	1.331537	2.06E-05	Up
	Abiotic	ATMG00670	1.32236	2.66E-05	Up
	Abiotic	ATMG00690	1.901881	2.19E-08	Up
	Abiotic	ATMG00730	2.050272	2.89E-09	Up
	Abiotic	ATMG00810	1.364922	3.26E-05	Up
	Abiotic	ATMG00940	1.053647	0.000543	Up
	Abiotic	ATMG00960	1.462321	9.98E-06	Up
	Abiotic	ATMG01000	1.240501	0.000127	Up
	Abiotic	ATMG01090	1.412848	1.22E-05	Up
	Abiotic	ATMG01120	1.769154	1.43E-07	Up
	Abiotic	ATMG01130	1.905809	1.57E-08	Up
	Abiotic	ATMG01170	1.573476	8.67E-07	Up
	Abiotic	ATMG01200	1.469969	7.66E-06	Up
	Abiotic	ATMG01220	1.018357	0.000994	Up
	Abiotic	ATMG01360	1.861423	3.73E-08	Up
	Abiotic	AT1G11740	-1.04173	1.37E-05	Down
	Abiotic	AT1G12070	-1.32533	7.48E-07	Down
	Abiotic	AT1G12805	-1.02017	0.000946	Down
	Abiotic	AT1G27020	-1.62365	1.18E-06	Down
	Abiotic	AT1G54740	-1.16015	0.000112	Down
	Abiotic	AT1G65390	-1.01725	0.000474	Down
	Abiotic	AT1G66465	-1.13657	0.00033	Down
	Abiotic	AT1G68720	-1.04295	3.97E-07	Down
	Abiotic	AT2G17850	-1.00697	0.000813	Down
	Abiotic	AT2G21650	-1.01887	0.000101	Down
	Abiotic	AT2G27380	-1.23377	0.000136	Down
	Abiotic	AT2G28180	-1.11248	0.000407	Down
	Abiotic	AT2G30230	-1.21109	3.18E-06	Down
	Abiotic	AT2G30670	-1.20016	0.00017	Down
	Abiotic	AT2G39570	-1.41109	4.41E-08	Down
	Abiotic	AT2G39820	-1.01043	0.000678	Down
	Abiotic	AT2G41250	-1.1101	5.42E-06	Down
	Abiotic	AT3G01970	-1.15556	3.82E-05	Down
	Abiotic	AT3G05150	-1.26648	6.80E-06	Down
	Abiotic	AT3G11920	-1.24199	0.000121	Down
	Abiotic	AT3G15356	-1.01925	0.000187	Down
	Abiotic	AT3G15620	-1.0347	8.26E-07	Down
	Abiotic	AT3G21351	-1.08922	5.23E-06	Down
	Abiotic	AT3G21530	-1.05696	1.51E-05	Down
	Abiotic	AT3G23550	-1.08302	0.000291	Down
	Abiotic	AT3G27785	-1.2688	4.84E-05	Down
	Abiotic	AT3G28170	-1.09789	0.000351	Down
	Abiotic	AT3G51910	-1.14832	0.000156	Down
	Abiotic	AT3G52510	-1.11043	0.00042	Down

Abiotic	AT3G54510	-1.63031	4.00E-07	Down
Abiotic	AT3G61198	-1.07293	5.07E-05	Down
Abiotic	AT4G11210	-1.09757	0.000284	Down
Abiotic	AT4G11460	-1.40491	3.18E-06	Down
Abiotic	AT4G14130	-1.31794	4.96E-07	Down
Abiotic	AT4G15680	-1.28703	2.69E-05	Down
Abiotic	AT4G16350	-1.04203	1.21E-05	Down
Abiotic	AT4G20450	-1.05845	0.000279	Down
Abiotic	AT4G25750	-1.01435	3.78E-05	Down
Abiotic	AT5G04190	-1.42268	2.71E-07	Down
Abiotic	AT5G10100	-1.3906	6.27E-06	Down
Abiotic	AT5G14000	-1.00087	0.000261	Down
Abiotic	AT5G14400	-1.2268	0.000132	Down
Abiotic	AT5G39660	-1.01027	2.15E-05	Down
Abiotic	AT5G53160	-1.05387	3.91E-07	Down
Abiotic	AT5G53980	-1.72352	3.65E-08	Down
Abiotic	AT5G55270	-1.03197	0.00069	Down
Abiotic	ATCG00120	-1.11198	0.00026	Down
Abiotic	ATCG00130	-1.16047	9.45E-05	Down
Abiotic	ATCG00150	-1.1315	0.000127	Down
Abiotic	ATCG00160	-1.1117	0.000406	Down
Abiotic	ATCG00170	-1.14322	0.000188	Down
Abiotic	ATCG00180	-1.13068	0.000147	Down
Abiotic	ATCG00340	-1.13883	0.000337	Down
Abiotic	ATCG00350	-1.15864	0.000138	Down
Abiotic	ATCG00360	-1.16438	0.000176	Down
Abiotic	ATCG00530	-1.52442	3.42E-06	Down
Abiotic	ATCG00540	-1.19412	0.000187	Down
Abiotic	ATCG00550	-1.02316	0.000956	Down
Abiotic	ATCG00600	-1.26264	9.34E-05	Down
Abiotic	ATCG00650	-1.09114	0.00053	Down
Abiotic	ATCG00660	-1.03167	0.000576	Down
Abiotic	ATCG00740	-1.09856	0.000479	Down
Abiotic	ATCG00770	-1.40354	7.59E-06	Down
Abiotic	ATCG00780	-1.14081	0.000125	Down
Abiotic	ATCG00800	-1.64287	4.21E-07	Down
Abiotic	ATCG00810	-1.26368	6.99E-05	Down
Abiotic	ATCG01020	-1.03471	0.000856	Down
Abiotic	ATCG01070	-1.04453	0.000767	Down
Abiotic	ATCG01090	-1.04604	0.000791	Down
Abiotic	ATCG01100	-1.04059	0.000545	Down
Abiotic	ATCG01130	-1.12274	0.00036	Down

Table S2. Gene ontology enrichment analysis of the DEGs under abiotic and biotic stresses.

Abiotic				Biotic			
**Term_type**	**GO number**	**Term**	**Query-item**	**Term_type**	**GO number**	**Term**	**Query-item**
Biological process	GO:0044249	cellular biosynthetic process	80	Biological process	GO:0051707	response to other organism	32

Biological process	GO:0009058	biosynthetic process	83	Biological process	GO:0009607	response to biotic stimulus	33
Biological process	GO:0009628	response to abiotic stimulus	84	Biological process	GO:0031323	regulation of cellular metabolic process	33
Biological process	GO:0042221	response to chemical stimulus	89	Biological process	GO:0051704	multi-organism process	34
Biological process	GO:0006950	response to stress	104	Biological process	GO:0010033	response to organic substance	37
Biological process	GO:0044260	cellular macromolecule metabolic process	106	Biological process	GO:0006952	defense response	43
Biological process	GO:0043170	macromolecule metabolic process	111	Biological process	GO:0042221	response to chemical stimulus	58
Biological process	GO:0050896	response to stimulus	134	Biological process	GO:0050794	regulation of cellular process	63
Biological process	GO:0044238	primary metabolic process	137	Biological process	GO:0050789	regulation of biological process	66
Biological process	GO:0044237	cellular metabolic process	159	Biological process	GO:0006950	response to stress	72
Biological process	GO:0008152	metabolic process	174	Biological process	GO:0065007	biological regulation	77
Biological process	GO:0009987	cellular process	190	Biological process	GO:0050896	response to stimulus	116
Molecular function	GO:0015077	monovalent inorganic cation transmembrane transporter activity	17	Molecular function	GO:0017076	purine nucleotide binding	30
Molecular function	GO:0022890	inorganic cation transmembrane transporter activity	17	Molecular function	GO:0016491	oxidoreductase activity	34
Molecular function	GO:0008324	cation transmembrane transporter activity	21	Molecular function	GO:0043169	ation binding	34
Molecular function	GO:0015075	ion transmembrane transporter activity	22	Molecular function	GO:0043167	ion binding	34
Molecular function	GO:0022891	substrate-specific transmembrane transporter activity	28	Molecular function	GO:0016301	kinase activity	37
Molecular function	GO:0022892	substrate-specific transporter activity	29	Molecular function	GO:0016772	transferase activity, transferring phosphorus-containing groups	37

Molecular function	GO:0022857	transmembrane transporter activity	31	Molecular function	GO:0003700	transcription factor activity	37
Molecular function	GO:0005215	transporter activity	36	Molecular function	GO:0030528	transcription regulator activity	38
Molecular function	GO:0016491	oxidoreductase activity	42	Molecular function	GO:0016787	hydrolase activity	52
Molecular function	GO:0003700	transcription factor activity	52	Molecular function	GO:0016740	transferase activity	70
Molecular function	GO:0030528	transcription regulator activity	54	Molecular function	GO:0005488	binding	163
Molecular function	GO:0003677	DNA binding	58	Molecular function	GO:0003824	catalytic activity	168
Cellular component	GO:0032991	macromolecular complex	55	Cellular component	GO:0009505	plant-type cell wall	8
Cellular component	GO:0016020	membrane	75	Cellular component	GO:0048046	apoplast	11
Cellular component	GO:0044444	cytoplasmic part	115	Cellular component	GO:0030312	external encapsulating structure	16
Cellular component	GO:0005737	cytoplasm	128	Cellular component	GO:0005618	cell wall	16
Cellular component	GO:0043231	intracellular membrane-bounded organelle	141	Cellular component	GO:0005576	extracellular region	17
Cellular component	GO:0043227	membrane-bounded organelle	141	Cellular component	GO:0031224	intrinsic to membrane	19
Cellular component	GO:0043229	intracellular organelle	147	Cellular component	GO:0044425	membrane part	24
Cellular component	GO:0043226	organelle	147	Cellular component	GO:0005886	plasma membrane	26
Cellular component	GO:0044424	ntracellular part	169	Cellular component	GO:0016020	membrane	63
Cellular component	GO:0005622	intracellular	173	Cellular component	GO:0012505	endomembrane system	70
Cellular component	GO:0044464	cell part	252	Cellular component	GO:0044464	cell part	212
Cellular component	GO:0005623	cell	252	Cellular component	GO:0005623	cell	212

Table S3. Identified transcription factors in response to biotic and abiotic stresses.

		Abiotic				Biotic		TF Family	Abiotic
TF Family Name	TF Locus Id	Protein Name	Gene Name , Synonym	Family Name	TF Locus Id	Protein Name	Gene Name , Synonym	AP2-EREBP	15
AP2-EREBP	At1g28370		ATERF11 , ERF11	AP2-EREBP	At2g38340			bHLH	2

AP2-EREBP	At1g74930		ORA47	AP2-EREBP	At1g21910			C2C2-CO-like	1
AP2-EREBP	At2g20880			bHLH	At5g56960	At-bHLH41		C2C2-Dof	2
AP2-EREBP	At2g38340			C2H2	At3g46080			C2H2	8
AP2-EREBP	At2g40340			C2H2	At3g46090		ZAT7, ZAT72	C3H	3
AP2-EREBP	At2g47520			C2H2	At3g28210		PMZ	CCAAT-HAP2	1
AP2-EREBP	At3g11020		DREB2, DREB2B	C2H2	At2g28710			G2-like	1
AP2-EREBP	At4g25480		CBF3, DREB1A	C3H	At1g63840			GRAS	1
AP2-EREBP	At4g25490		CBF1, DREB1B	CCAAT-HAP3	At5g47670		L1L, NF-YB6	Homeobox	2
AP2-EREBP	At4g28140			HSF	At4g36990		HSF	5	
AP2-EREBP	At5g05410		DREB2, DREB2A	MYB	At1g74080	AtMYB122	ATMYB122, MYB122	MYB	2
AP2-EREBP	At5g25390		SHINE2, SHN2	MYB	At1g57560	AtMYB50	AtMYB50	NAC	6
AP2-EREBP	At5g51990		CBF4, DREB1D	MYB	At2g32460	AtMYB101	ATM1, ATMYB101, MYB101	RAV	1
AP2-EREBP	At5g61890			NAC	At3g44350		anac061	REM	
AP2-EREBP	At5g64750		ABR1	NAC	At1g34180		anac016	Trihelix	
bHLH	At3g22100	At-bHLH117		NAC	At2g43000		anac042	WRKY	1
bHLH	At5g57150	At-bHLH35		NAC	At5g22380		anac090		
C2C2-CO-like	At3g21890			NAC	At1g02220		ANAC003		
C2C2-Dof	At4g00940			NAC	At5g18270		ANAC087		
C2C2-Dof	At5g39660		CDF2	REM	At3g46770		REM22		
C2H2	At1g27730		STZ, ZAT10	Trihelix	At2g38250				
C2H2	At1g66500			WRKY	At4g11070	At-WRKY41	AtWRKY41, WRKY41		
C2H2	At3g19580		AZF2	WRKY	At2g21900	At-WRKY59	AtWRKY59, WRKY59		
C2H2	At3g28210		PMZ	WRKY	At1g66600	At-WRKY63	AtWRKY63, WRKY63		
C2H2	At5g04340		C2H2, CZF2, ZAT6	WRKY	At5g64810	At-WRKY51	atwrky51, WRKY51		

C2H2	At5g43620			WRKY	At5g13080	At-WRKY75	AtWRKY75, WRKY75
C2H2	At5g59820		RHL41, ZAT12	WRKY	At4g22070	At-WRKY31	AtWRKY31, WRKY31
C2H2	At3g05150			WRKY	At1g68150	At-WRKY9	AtWRKY9, WRKY9
C3H	At3g15740			WRKY	At5g15130	At-WRKY72	AtWRKY72, WRKY72
C3H	At4g33940			WRKY	At2g40740	At-WRKY55	AtWRKY55, WRKY55
C3H	At5g38895			WRKY	At2g46400	At-WRKY46	AtWRKY46, WRKY46
CCAAT-HAP2	At1g54160		NF-YA5, NFYA5	WRKY	At5g24110	At-WRKY30	AtWRKY30, WRKY30
G2-like	At3g46640		LUX, PCL1	WRKY	At4g01720	At-WRKY47	AtWRKY47, WRKY47
GRAS	At4g17230		SCL13	WRKY	At5g26170	At-WRKY50	ATWRKY50, WRKY50
Homeobox	At3g03260		HDG8	WRKY	At5g01900	At-WRKY62	AtWRKY62, WRKY62
Homeobox	At5g53980		ATHB52	WRKY	At3g01970	At-WRKY45	AtWRKY45, WRKY45
HSF	At2g26150		ATHSFA2, HSFA2				
HSF	At4g11660						
HSF	At4g36990						
HSF	At5g03720		AT-HSFA3, HSFA3				
HSF	At3g51910		AT-HSFA7A, HSFA7A				
MYB	At1g56650	AtMYB75					
MYB	At4g21440	AtMYB102	ATM4, ATMYB102				
NAC	At1g01720		ANAC002, ATAF1				

NAC	At1g32870		ANAC013, ANAC13
NAC	At1g52890		ANAC019
NAC	At1g69490		ANAC029, ATNAP, NAP
NAC	At4g27410		ANAC072, RD26
NAC	At5g22290		anac089
RAV	At3g11580		
WRKY	At3g01970	At-WRKY45	At-WRKY45, WRKY45

Table S4. miRNAs associated with the DEGs were discovered using the computational algorithm psRNATarget server.

	**Abiotic**	**Biotic**
miRNA		miRNA_Acc.
ath-miR858	8	ath-miR172
ath-miR5665	6	ath-miR414
ath-miR5658	4	ath-miR5021
ath-miR395	3	ath-miR5633
ath-miR162	2	ath-miR5658
ath-miR413	2	
ath-miR5648	2	
ath-miR838	2	
ath-miR161	1	
ath-miR3440	1	
ath-miR398	1	
ath-miR400	1	
ath-miR407	1	
ath-miR414	1	
ath-miR4221	1	
ath-miR447	1	
ath-miR5023	1	
ath-miR5640	1	
ath-miR5653	1	
ath-miR5654	1	
ath-miR5657	1	
ath-miR777	1	
ath-miR8166	1	
ath-miR847	1	
ath-miR865	1	
